# The *Francisella* Type VI Secretion System

**DOI:** 10.3389/fcimb.2018.00121

**Published:** 2018-04-23

**Authors:** Daniel L. Clemens, Bai-Yu Lee, Marcus A. Horwitz

**Affiliations:** Division of Infectious Diseases, Department of Medicine, University of California, Los Angeles, Los Angeles, CA, United States

**Keywords:** tularemia, *Francisella tularensis*, *Francisella* pathogenicity island, CryoElectron microscopy, atomic model, review

## Abstract

*Francisella tularensisis* subsp. *tularensis* is an intracellular bacterial pathogen and the causative agent of the life-threatening zoonotic disease tularemia. The *Francisella* Pathogenicity Island encodes a large secretion apparatus, known as a Type VI Secretion System (T6SS), which is essential for *Francisella* to escape from its phagosome and multiply within host macrophages and to cause disease in animals. The T6SS, found in one-quarter of Gram-negative bacteria including many highly pathogenic ones, is a recently discovered secretion system that is not yet fully understood. Nevertheless, there have been remarkable advances in our understanding of the structure, composition, and function of T6SSs of several bacteria in the past few years. The system operates like an inside-out headless contractile phage that is anchored to the bacterial membrane via a baseplate and membrane complex. The system injects effector molecules across the inner and outer bacterial membrane and into host prokaryotic or eukaryotic targets to kill, intoxicate, or in the case of *Francisella*, hijack the target cell. Recent advances include an atomic model of the contractile sheath, insights into the mechanics of sheath contraction, the composition of the baseplate and membrane complex, the process of assembly of the apparatus, and identification of numerous effector molecules and activities. While *Francisella* T6SS appears to be an outlier among T6SSs, with limited or no sequence homology with other systems, its structure and organization are strikingly similar to other systems. Nevertheless, we have only scratched the surface in uncovering the mysteries of the *Francisella* T6SS, and there are numerous questions that remain to be answered.

## Introduction

*Francisella tularensis* subsp. *tularensis* is a Gram-negative bacterium that causes a serious and potentially fatal zoonotic infection, tularemia, in animals and humans (Ellis et al., 2002). *F. tularensis* has a relatively broad host-range and is capable of multiplying intracellularly in insects as well as in a wide range of mammals, including rabbits, rodents, beavers, and man. For mammals, *F. tularensis* is the most infectious bacterial pathogen known; the LD_50_ in mice for a subcutaneous inoculation of the virulent SCHU S4 strain is 1–4 organisms (Bell et al., 1955), and in humans, as few as 10 organisms delivered subcutaneously or 25 organisms delivered by inhalation can lead to life threatening infection (Saslaw et al., 1961a,b). Because of its high infectivity and lethality, the ease with which it can be cultured and dispersed, and the history of its use as a bioweapon, it is considered a potential agent of bioterrorism and is classified as a Tier 1 Select Agent. This has led to renewed interest and investigation of its cell biology and the pathogenic mechanisms underlying its remarkable infectivity.

## Intracellular life cycle of *F. tularensis*

**Figure 1:**
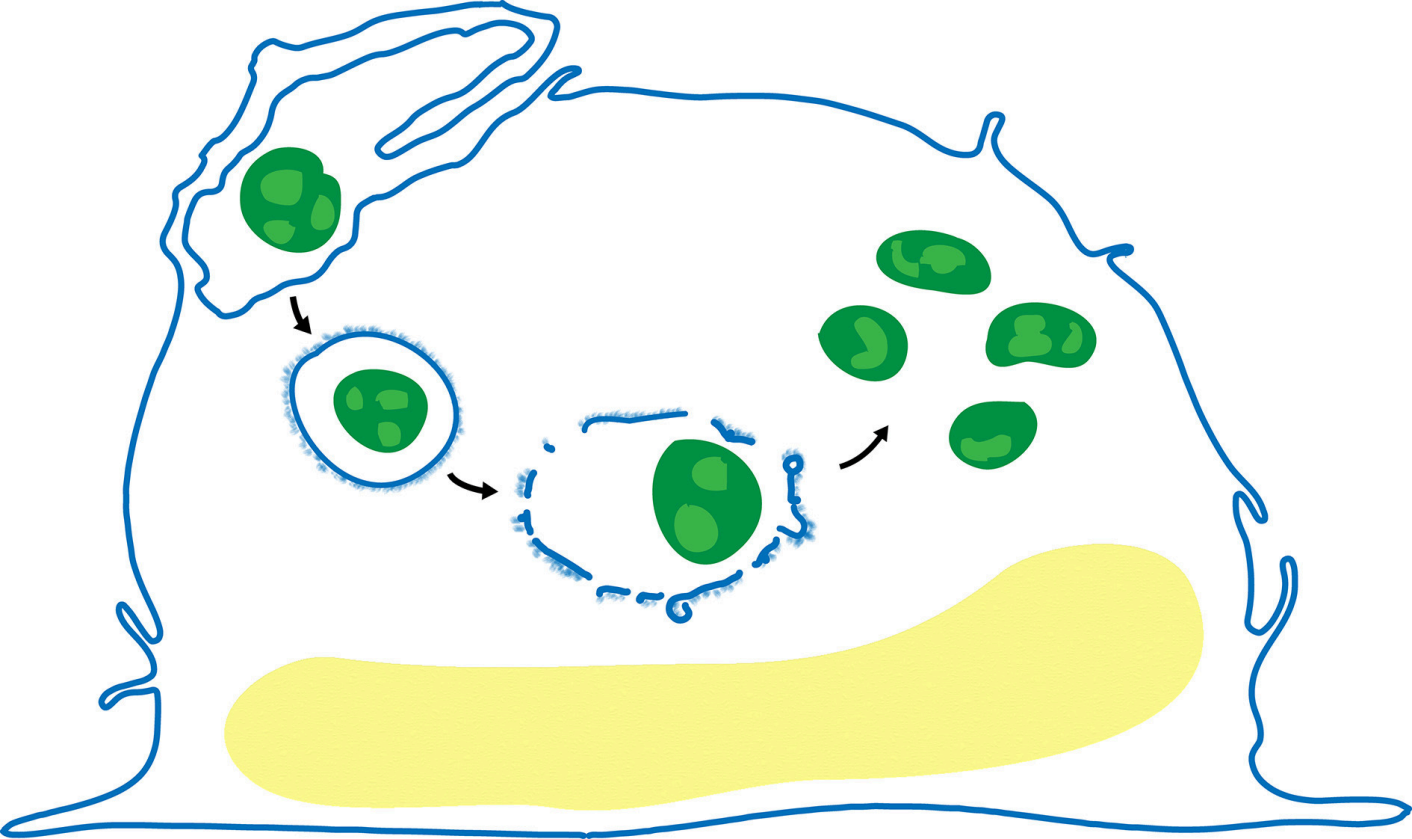
Life cycle of *F. tularensis* in human macrophages. After uptake by looping phagocytosis (upper left), the bacteria (green) reside in a membrane bound vacuole that often acquires a densely staining fibrillar coat (post first arrow), which subsequently forms blebs and vesicles (post second arrow), and disintegrates. The bacteria escape the phagosome and replicate freely in the cytosol (post third arrow).

Although *F. tularensis* infection has been demonstrated in many host cells, including alveolar epithelial cells, neutrophils, and hepatocytes, macrophages are infected early in infection and are important both as a major site of bacterial replication and in host defense against infection. We have shown that the bacteria are internalized by macrophages via a novel mechanism–looping phagocytosis (Clemens et al., 2005; Clemens and Horwitz, 2007; Figure 1), and that the O-antigen polysaccharide plays a role in the morphology of this process (Clemens et al., 2012). Following uptake, *F. tularensis* resides in a membrane-bound vacuole that acquires early endosomal markers, but resists maturation, as evidenced by its failure to fuse with secondary lysosomes and its only limited acquisition of cathepsin D and lysosome-associated membrane glycoproteins. Ultrastructurally, the *F. tularensis* phagosome acquires a unique, densely staining fibrillar coat that forms blebs and vesicles and subsequently fragments, with escape of the bacterium into the cytosol, where it replicates freely (Golovliov et al., 2003; Clemens et al., 2004; Clemens and Horwitz, 2007; Chong and Celli, 2010; Figure 1). *F. tularensis* subsp. *tularensis* (*F. tularensis*) is genetically closely related to the attenuated vaccine strain *F. tularensis* subsp. *holarctica* Live Vaccine Strain (LVS) and to *F. tularensis* subsp*. novicida* (also classified and hereafter referred to as *F. novicida*; Johansson et al., 2010) and it shares with them the same intracellular life-style. After extensive replication within the host cell, the bacteria induce apoptosis or pyroptosis, culminating in release of bacteria that can initiate another round of infection in host cells (Lai et al., 2001; Mariathasan et al., 2005) or spread from cell to cell by way of trogocytosis (Bourdonnay and Henry, 2016; Steele et al., 2016).

The host cell does have innate defenses that come into play. Macrophage guanylate binding proteins act downstream of Type I interferon receptors to bind to *F. tularensis*, facilitating lysis of the bacteria and release of bacterial DNA, which in turn activates the AIM2 inflammasome and Caspase 1, IL-1β, and IL-18, leading to macrophage cell death and helping to control the infection (Henry et al., 2007; Weiss et al., 2007b; Man et al., 2015; Meunier et al., 2015).

Checroun et al. have shown, in mouse bone marrow-derived macrophages, that at late times after infection (> 20 h) a large percentage of *F. tularensis* LVS enter double-membraned, LC3-positive autophagic vacuoles (termed *Francisella*-containing vacuoles, FCVs) that are acidified, stain positively for LAMP-1 and cathepsin D and fuse with secondary lysosomes (Checroun et al., 2006). It is intriguing that the *F. tularensis* within the FCVs do not escape from these compartments via their Type VI Secretion System (T6SS) apparatus as they do from their phagosome post ingestion. These autophagic FCVs may reflect an aspect of host control of the intracellular infection, rather than a feature of the *F. tularensis* intracellular life cycle that benefits the bacterium, since induction of autophagy promotes eradication of infection (Chiu et al., 2009).

## The Francisella Pathogenicity Island

**Figure 2:**
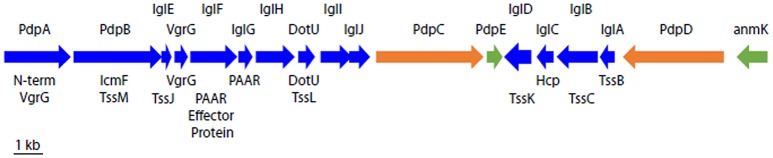
Schematic organization of the T6SS gene cluster on the FPI. Names of *F. novicida* gene products are shown above and names of the corresponding canonical T6SS gene products (where known) are shown below. Gene products shown in blue are required for growth in macrophages and for virulence in animals; those in orange are required for full virulence in animals (Weiss et al., 2007a) but not for growth in macrophages; and those in green are not required for growth in macrophages or for virulence in animals (Bröms et al., 2010).

Gray et al. (2002) used transposon mutagenesis to identify five genetic loci in *F. novicida* whose disruption led to impaired intracellular growth in macrophages: *iglA, iglB, iglC, iglD*, and *clpB*. While the induction of IglC in the intramacrophage environment was appreciated from work by Golovliov et al. (1997) the relation of these genes to the gene clusters of other bacteria and their role in a secretion system was not recognized at this time. The existence of the Francisella Pathogenicity Island (FPI) was first reported by Nano et al. (2004), who described a large cluster of genes on an island of the *F. novicida* chromosome with a relatively low GC content that is required for intracellular growth in macrophages and for virulence in mice. This ~30-kb island encodes 18 genes, 14 of which have been shown to be essential for growth in macrophages; all, except for *pdpE* and *anmK*, are required for full virulence in mice (Figure 2; Bröms et al., 2010).

Because some proteins encoded on the FPI (such as VgrG and DotU) show clear homology to the core components of T6SSs of other Gram-negative bacteria, it was proposed that the FPI encoded a T6SS. Bioinformatic analysis of IglA and IglB suggested that they could be components of a T6SS apparatus, and de Bruin et al. showed that IglA was expressed as a soluble cytoplasmic protein under control of the MglA and MglB global regulators, with expression markedly increasing after macrophage infection, and that IglA expression was essential to intramacrophage growth of *F. novicida*. (de Bruin et al., 2007). While most proteins of the FPI have little or no sequence homology to T6SS proteins of other bacteria, for those whose structure has been determined to date, the proteins have shown striking structural homology to other T6SSs. For example, we have recently shown by CryoEM that the FPI proteins IglA and IglB assemble into long cylinders with strong structural homology to the sheaths of contractile phage tails (myophages), R-type pyocins, and the contractile sheaths of other bacterial T6SSs, despite the absence of sequence homology (Clemens et al., 2015).

There are two copies of the FPI present in *F. tularensis* and *F. tularensis* subsp. *holarctica* LVS, while *F. novicida* has a single copy (Bröms et al., 2010); additionally, *F. novicida* possesses a related genomic island named *Francisella novicida* island (Rigard et al., 2016). Because *F. novicida* has only a single copy of the FPI and is of low virulence for humans, it has served as a more tractable subspecies for study and it is widely used for investigations of the FPI.

## Type VI secretion systems

Type VI Secretion Systems (T6SSs) are recently identified large nanomachines encoded on gene clusters that function like an inside-out contractile phage tail to inject effector molecules across the inner and outer bacterial membrane and into host prokaryotic or eukaryotic targets in order to kill, intoxicate, or in the case of *Francisella*, hijack the target cell (Bingle et al., 2008; Cianfanelli et al., 2016b). T6SSs are very widespread among bacteria, being found in one quarter of sequenced Gram-negative bacteria, including many that are important plant, animal, and human pathogens (Bingle et al., 2008; Boyer et al., 2009). T6SSs resemble, and are thought to be evolutionarily related to, other Contractile Injection Systems (CISs): myophages (Leiman and Shneider, 2012), R-type pyocins (Ge et al., 2015), anti-feeding phage (Afp) (Heymann et al., 2013), and metamorphosis associated structures (MACs) (Shikuma et al., 2014). However, these contractile injection systems have undergone extensive divergence so that sequence homologies are often limited, and it is unclear which system evolved first. The T6SS differs from other CISs in that it is the only one that contracts inside the organism (Böck et al., 2017). The T6SS remains within the intact bacterium when the apparatus contracts and injects its effectors across the bacterial inner and outer membranes and into the target cell. All other CISs, such as myophages, Afps, and MACs, are designated “eCISs” to indicate that they contract in the extracellular space following their release from the bacterium in which they were assembled (Böck et al., 2017).

CISs have in common several key components: a long contractile sheath, a tube that fits within the sheath tipped with a central spike/effector protein complex that is propelled by contraction of the sheath, and a baseplate complex (Leiman and Shneider, 2012). In addition to these components, T6SSs also include a membrane complex that anchors the baseplate to the membrane and allows passage of the central spike/effector complex and tube without compromise to the integrity of the host bacterial inner and outer membranes (Ho et al., 2014).

Canonical T6SSs have in common 13 conserved core subunits (designated “**T**ype **s**ix **s**ecretion subunits, TssA–TssM, Table 1) encoded on gene clusters that assemble to form the apparatus. The discovery and general characteristics of the T6SS have been the subject of several excellent reviews (Ho et al., 2014; Basler, 2015; Cianfanelli et al., 2016b; Hood et al., 2017). The first clue to the existence of this new secretion system was the observation that Haemolysis coregulated protein (Hcp), which lacks a signal sequence, was secreted by *Vibrio cholerae* (Williams et al., 1996). In 2003, Bladergroen et al. (2003) identified a gene cluster essential for secretion in *Rhizobium leguminosarum* that encoded what subsequently became known as a T6SS. They showed that this gene cluster influenced the host range of the bacterium and hypothesized that it was involved in protein secretion. Similar gene clusters were identified in many other bacteria, including *V. cholerae*, and observed to include genes homologous to the Type 4 secretion membrane protein gene, *icmF* (Das and Chaudhuri, 2003). Evidence for the role of a similar gene cluster in virulence of the fish pathogen *Edwardsiella tarda* came from the work of Rao et al., who used transposon mutagenesis and proteomic analysis to identify five proteins important to *E. tarda* pathogenesis (Rao et al., 2004). While three of the five proteins were homologous with T3SS effector proteins, Rao et al. recognized that the other two, named EvpA and EvpC, were encoded with in a cluster of 8 genes (*evpA-H*) similar to those described for *R. leguminosarum, V. cholerae*, and other bacteria. Rao et al. noted that EvpA and EvpB show 25 and 30% identity to *Francisella* IglA and IglB, respectively, that EvpC is homologous with Hcp, and that secretion of EvpC is blocked by disruption of either EvpA or EvpB, and they hypothesized that secretion of EvpC was not via the T3SS. Pukatzki et al. showed that the gene cluster in *V. cholerae* was essential for secretion of Hcp and VgrG (which also lacks a signal sequence) into the culture supernatant fluid and for the capacity of *V. cholerae* to kill *Dictyostelium discoideum* and J774 macrophages in a contact-dependent fashion (Pukatzki et al., 2006). Because addition of concentrated *V. cholerae* culture supernatants containing Hcp and VgrG proteins caused no cytotoxicity to *D. discoideum* amoebae, Pukatzki et al. proposed that the system was triggered by bacterium-eukaryotic cell contact and functioned to inject the secreted effector proteins into the eukaryotic cell cytosol; they named the system the Type 6 Secretion System (Pukatzki et al., 2006). A structural counterpart to these gene clusters was discovered using fluorescence microscopy and electron cryotomography (ECT) in intact *V. cholerae*, with visualization of the structure in both its extended and contracted states (Basler et al., 2012).

**Table 1:** Essential T6SS proteins and their orthologues in *Francisella* and T4 Phage.

**T6SS**	**Fn T6SS**	**T4 phage**	**Function**
TssA	?	gp3/gp15	Assembly Chaperone and Tube/Sheath Cap
TssBC	IglAB	gp18	Sheath
Hcp	IglC	gp19	Tube
(Hcp)	(IglC)	gp48	Tube/Baseplate
(Hcp)	(IglC)	gp54	Tube/Baseplate
VgrG	VgrG and PdpA	gp5 and gp27	Spike complex
PAAR Protein	IglG (?)	gp5.4	Spike tip
PAAR Associated Effector Protein	IglF (?)	gp5.4	Effector
TssF	?	gp6	Baseplate
TssG	?	gp7/gp53	Baseplate
TssE	?	gp25	Baseplate
TssK	IglD	gp10^*^ (Siphophage RBP)	Baseplate
TssJ	IglE	None	Membrane complex
TssL/DotU	DotU	None	Membrane complex
TssM/IcmF	PdpB	None	Membrane complex
ClpV	ClpB	None	Sheath disassembly

* *TssK has no structural homolog in T4 phage baseplate, but TssK appears to occupy the same position in the baseplate as domain IV of gp10 (Nazarov et al., 2018). TssK has structural homology with Siphophage Receptor Binding Protein (RBP, Nguyen et al., 2017)*.

## Role of the *Francisella* T6SS in phagosomal escape and intracellular replication

T6SSs play different roles in the biology of their host organisms depending upon the life style of that organism. In the case of extracellular bacterial pathogens, such as *V. cholerae, Pseudomonas aeruginosa*, and enteropathogenic *E. coli*, the T6SSs primarily function in interbacterial warfare. For example, Basler *et al*. have shown that, in the case of *P. aeruginosa*, T6SS activity first occurring in prey species *V. cholerae* and *Acinetobacter baylyi* triggers reciprocal *P. aeruginosa* T6SS formation at the point of contact with the prey's T6SS with the result that the prey bacterium is killed in a “tit-for-tat” fashion (Basler et al., 2013). Some bacteria have several T6SSs, which may be regulated differently and secrete effectors with diverse functions and distinct target specificities (Bingle et al., 2008; Schwarz et al., 2010; Journet and Cascales, 2016). For example, *P. aeruginosa* has three T6SSs (H1-, H2-, and H3-T6SS). While H1-T6SS secretes toxins to counter other Gram-negative bacteria with T6SSs, H2-, and H3-T6SSs act on both prokaryotic and eukaryotic cells (Sana et al., 2016). In the case of the intracellular bacterial pathogen *F. tularensis* and other *Francisella*, the T6SS is required for phagosomal escape, intracytoplasmic replication in host cells, and virulence in animals (Lindgren et al., 2004; Nano et al., 2004; de Bruin et al., 2007; Bröms et al., 2010). Our structure-based mutagenesis studies of the *F. novicida* T6SS have demonstrated that mutations in the sheath proteins IglA and IglB that interfere with contraction of the sheath block T6SS secretion, phagosomal escape, and replication in human macrophage-like cells (Clemens et al., 2015). The T6SS of *Candidatus Amoebophilus asiaticus*, an obligate intracellular bacterial symbiont of amoebae, may serve a similar function in promoting phagosome escape for this organism (Böck et al., 2017).

All FPI genes that are required for phagosome escape and intracellular replication in macrophages are also required for full virulence in animals (Figure 2). However, while *pdpC* (Long et al., 2013) and *pdpD* (Ludu et al., 2008; Brodmann et al., 2017) are essential for full virulence in animals, their absence results in relatively minor defects in growth in macrophages. Virulence in animals may be highly sensitive to defects in intramacrophage growth, or perhaps PdpC and PdpD impact the innate immune response, which plays a greater role *in vivo* in animals than *in vitro* in macrophage cell culture. Interestingly, PdpD is present in *F. novicida* and the highly virulent Type A *F. tularensis*, but not in the less lethal Type B *F. tularensis* (Ludu et al., 2008).

## T6SS classification

**Figure 3:**
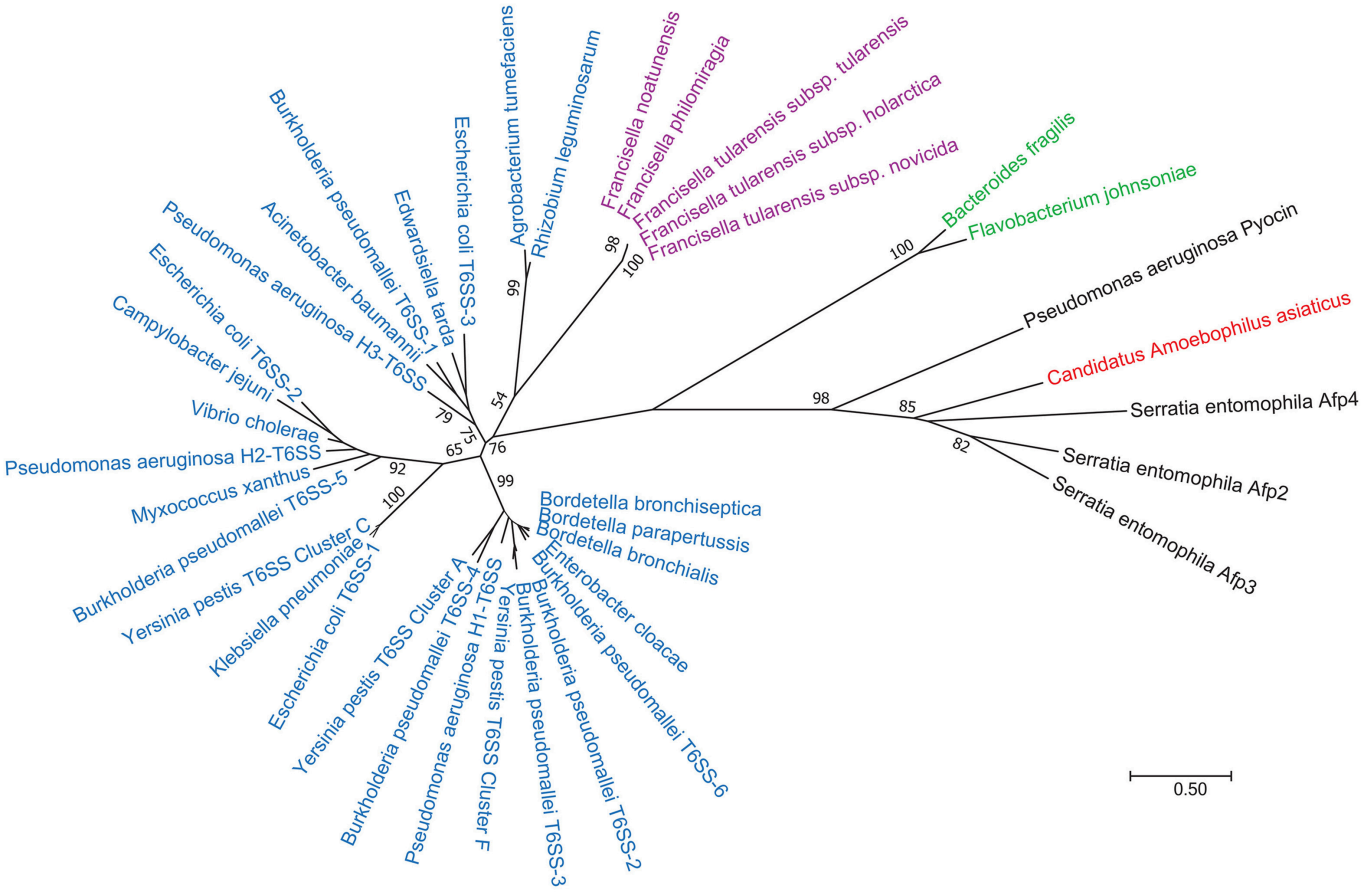
Phylogenetic tree of the T6SS and eCIS large sheath subunit. The Maximum Likelihood method, based on the JTT matrix-based model (Jones et al., 1992) was used to infer evolutionary history of the T6SS large sheath subunit based on amino acid sequences. The tree with the highest log likelihood (−8830.54) is shown. The numbers next to the branches indicate the percentage of trees in which the associated taxa clustered together in the bootstrap test (1,000 replicates). Initial trees for the heuristic search were obtained automatically by applying Neighbor-Join and BioNJ algorithms to a matrix of pairwise distances estimated using a JTT model, and then selecting the topology with superior log likelihood value. The tree is drawn to scale, with branch lengths measured in the number of substitutions per site. The scale bar at lower right indicates a distance corresponding to 0.5 substitutions per site. Members of T6SS class 1 are colored blue, T6SS class 2 are colored purple, T6SS class 3 are colored green, T6SS class 4 are colored red, and eCISs are shown in black. The analysis involved 39 amino acid sequences. All positions containing gaps and missing data were eliminated. There were a total of 185 positions in the final dataset. Evolutionary analyses were conducted in MEGA7 (Kumar et al., 2016).

T6SSs have been classified into three categories (T6SS^i−iii^, Table 2 and Figure 3) based on their genetic make-up, with T6SS^i^ encompassing canonical T6SSs of *Vibrio, Pseudomonas*, and Enteropathogenic *E. coli*; T6SS^ii^ representing *Francisella*; and T6SS^iii^ comprised of *B. fragilis and Flavobacterium johnsoniae* (Russell et al., 2014b). Figure 3 shows the genetic relatedness of the large sheath unit (IglB, VipB, TssC) in bacteria with T6SS and, for comparison, the sheath subunits of Afp and R-pyocin. Similar maps have been made using the large sheath subunit (Rao et al., 2004), small sheath subunit (IglA, VipA, TssA) (Schwarz et al., 2010), and the putative baseplate protein TssF (Journet and Cascales, 2016). While it is clear that this classification system reflects genetic and evolutionary relatedness, it is unclear whether these 3 categories correlate with structural or functional differences in the systems. For example, while the *F. tularensis* T6SS has been classified genetically as an outlier among T6SSs, every component of its apparatus whose structure has been determined thus far has shown striking structural homology to the structures of the canonical T6SS. On the other hand, a new T6SS^iv^ category (Table 2 and Figure 3) has recently been described in *Ca. A. asiaticus* (Böck et al., 2017) which is both genetically and structurally closer to eCIS, although it functions intracellularly like T6SS^i−iii^.

**Table 2:** Classification of T6SSs. T6SS^i−ii^ and T6SS^iii−iv^ belong to Gram-negative bacteria of taxonomically distinct subgroups.

**Organism**	**Phylum**	**Class**	**T6SS Category**
*Vibrio cholerae*	Gammaproteobacteria	Gammaproteobacteria	T6SS^i^
*Pseudomonas aeruginosa*	Gammaproteobacteria	Gammaproteobacteria	T6SS^i^
*Francisella tularensis*	Gammaproteobacteria	Gammaproteobacteria	T6SS^ii^
*Bacteroides fragilis*	Bacteroidetes	Bacteroidetes	T6SS^iii^
*Flavobacterium johnsoniae*	Bacteroidetes	Flavobacteriia	T6SS^iii^
*Ca. Amoebophilus asiaticus*	Bacteroidetes	Cytophagis	T6SS^iv^

## Identification of environmental factors that induce assembly of the *Francisella* T6SS

Golovliov et al. identified IglC as an *F. tularensis* protein that was induced by bacteria within macrophages or under oxidative stress (Golovliov et al., 1997); subsequently IglC was shown to be required for intracellular growth in macrophages (Lai et al., 2004) before its role in the T6SS was known. Other studies reported that FPI genes *iglA, iglB, iglC, pdpA*, and *pdpD* [and also *clpB*, a T6SS-related gene outside the FPI (Brodmann et al., 2017)] show increased expression in the intramacrophage environment (Wehrly et al., 2009). Activation of the stringent response by growth of *F. tularensis* SCHU S4 in culture medium with serine hydroxamate was recently shown to increase the expression of multiple FPI genes, including both copies of *iglA, iglB, iglC, iglD, pdpA*, and *pdpD* (Murch et al., 2017). Iron restriction, a condition associated with the intracellular environment, has been shown to increase expression of several FPI proteins, including IglC, IglD, IglA, and PdpB, and putative Fur boxes have been identified in front of *pdpB* and *iglC* (Deng et al., 2006). While oxidative stress, iron deprivation, and stringent response increase expression of FPI proteins, it has not been reported whether these maneuvers lead to increased T6SS assembly or secretion.

Whereas many T6SSs, such as those of *Vibrio*, the *Pseudomonas* H2-T6SS (Haapalainen et al., 2012; Decoin et al., 2014) and *Burkholderia*, show a basal level of secretion in broth culture, we have observed that *F. tularensis* LVS and *F. novicida* do not (Clemens et al., 2015). This initially hampered structural and functional studies of the *Francisella* T6SS and the identification of additional proteins secreted by the system.

**Figure 4:**
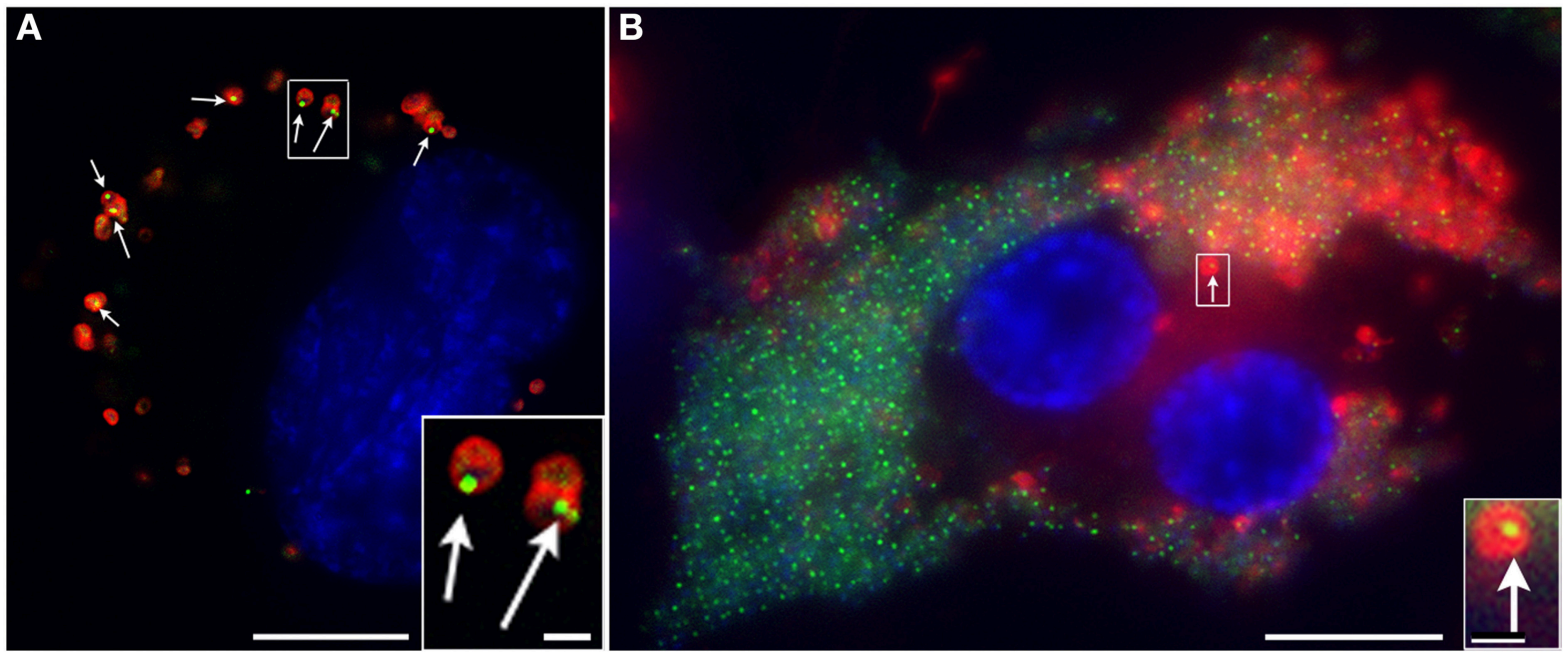
*F. novicida* expressing sfGFP-tagged IglA form intensely fluorescent structures after uptake by macrophages, with 10% doing so at 15 min of infection **(A)** and 70% at 22 h of infection **(B)**. *F. novicidia* are stained with a red fluorescent antibody; host and bacterial DNA are stained blue with DAPI; and arrows indicate bacteria shown at higher magnification in the insets. Scale bars 10 μm (insets 1 μm). Reproduced with permission from Clemens et al. (2015).

**Figure 5:**
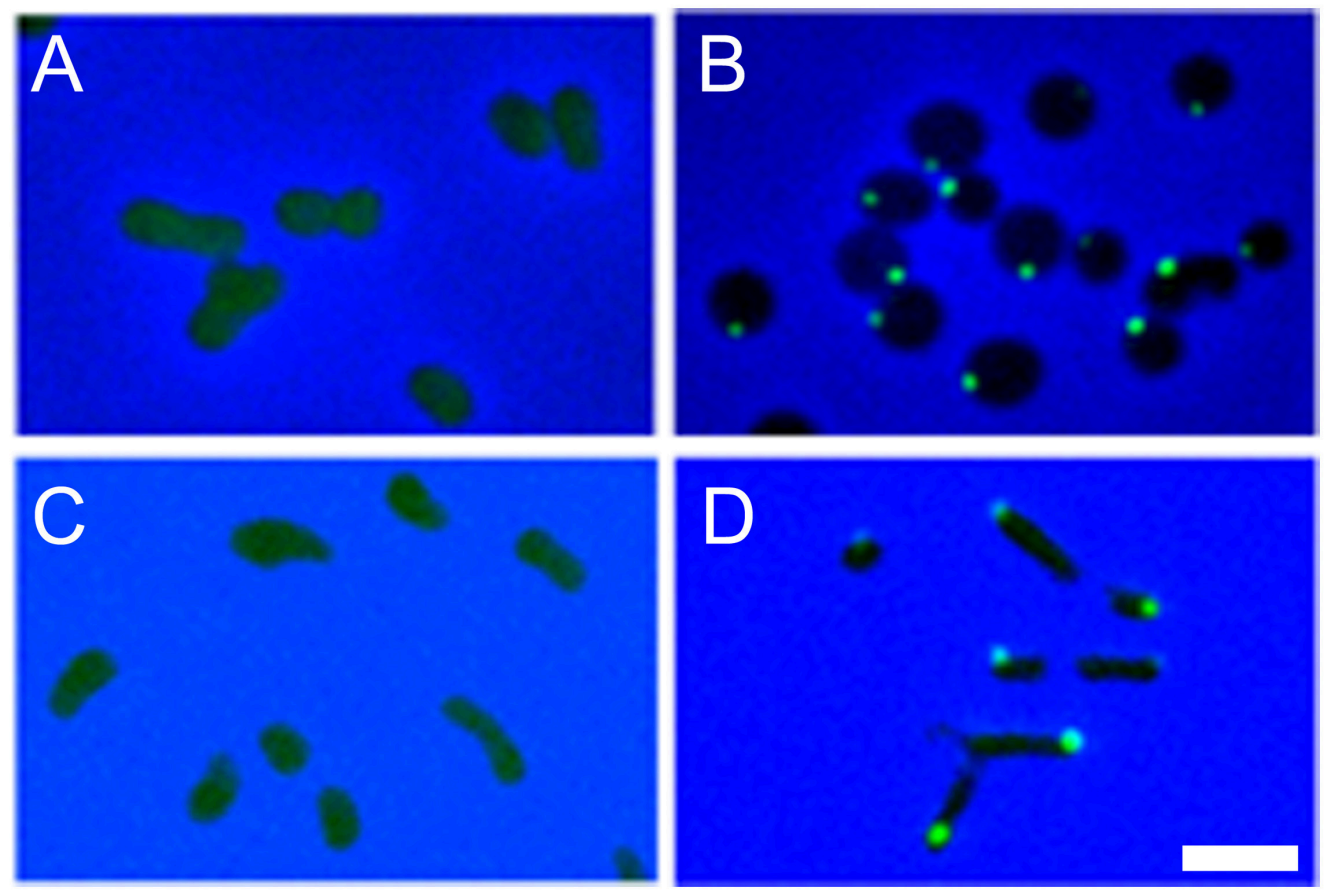
*F. novicida* expressing IglA-sfGFP form fluorescent structures after placement beneath a glass coverslip **(A,B)** and growing in liquid culture medium with high KCl concentration **(C,D)**. The bacteria initially show only a diffuse fluorescence when placed beneath a coverslip **(A)**, but after 4 h at room temperature, the majority of the bacteria exhibit intensely fluorescent structures **(B)**. Bacteria exhibit only diffuse fluorescence when grown in standard broth culture **(C)**, but form fluorescent foci when grown in culture medium with 5% KCl **(D)**. Scale bar, 2 μm. Reproduced with permission from Clemens et al. (2015).

Preparation of *F. novicida* expressing IglA-sfGFP enabled us to search for *in vitro* conditions that induced formation of fluorescent structures within the bacteria. We found that the bacteria were not fluorescent when grown in standard broth culture, but that they rapidly acquired fluorescent foci after uptake by macrophages (Clemens et al., 2015; Figure 4). In addition, we found that bacteria placed on a microscope slide beneath a glass coverslip initially lacked fluorescent foci, but with time developed them (Figure 5). This was not due to evaporation and concentration of the culture medium, as bacteria continued to form fluorescent foci even when the coverslip was sealed with silicone. The nature of the coverslip stimulus sensed by *F. novicida* is unclear and may reflect a combination of factors, such as mechanical pressure from the coverslip and a decrease in oxygen tension. The use of gas permeable plastic coverslips instead of glass coverslips delays, but does not prevent, the formation of the fluorescent foci. We hypothesized that the induction of the T6SS within macrophages reflects a response by bacteria to conditions in the host cytoplasm that differ from those in standard broth culture medium. This prompted us to examine whether increasing the concentration of KCl would induce formation of fluorescent foci or T6SS secretion. We found that fluorescent foci did form in TSBC broth culture with 2.5 or 5% KCl (Figure 5), but not at lower concentrations, and that the formation of the fluorescent foci was accompanied by secretion of VgrG and IglC (Clemens et al., 2015).

**Figure 6:**
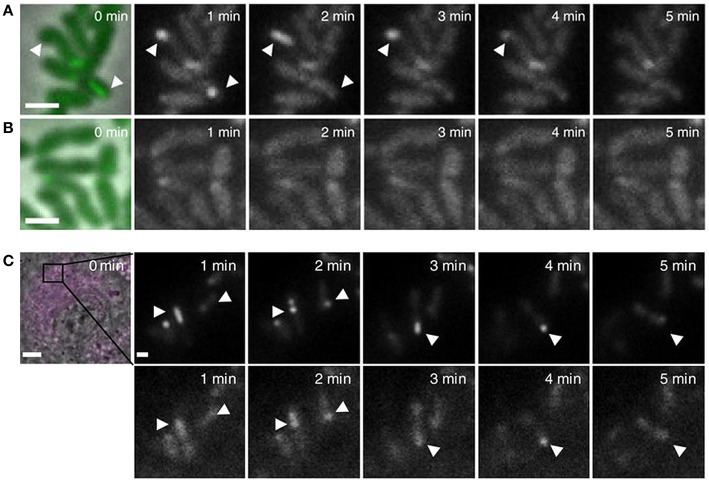
**(A,B)** Live fluorescence microscopy shows dynamic formation and disassembly of IglA-sfGFP fluorescent structures in wild-type *F. novicida* **(A)** but not Δ*pdpB F. novicida* **(B)**. Arrow heads indicate positions of fluorescent sheath assembly, contraction, and disassembly. Reproduced with permission from Brodmann et al. (2017). **(C)** Time-lapse images of unprimed wild-type BMDMs infected with *F. novicida* expressing IglA-sfGFP ClpB-mCherry2 for 1 h. First image shows merged phase contrast, GFP and mCherry channels with a 30 × 30 μm field of view. Scale bar, 5 μm. Close ups show GFP channel (upper panels) and mCherry channel (lower panels). Close ups show 5 × 5 μm fields of view. Scale bar, 1 μm. Arrowheads indicate positions of T6SS sheath assembly, contraction and location of sheath after contraction. Reproduced with permission from Brodmann et al. (2017).

While we did not observe in *F. novicida* the dynamic formation, contraction and disassembly of the T6SS described for *E. coli* or *V. cholerae*, this has recently been observed (Brodmann et al., 2017). Using improved instrumentation and image collection, Brodmann et al. (2017) have shown that *F. novicida* expressing IglA-sfGFP form dynamic sheaths that assemble, contract, and disassemble in a fashion similar to what has been shown for *V. cholerae* and *E. coli* (Figure 6). Dynamic assembly was observed both for free bacteria resuspended in phosphate buffered saline (Figure 6A) and for bacteria within mouse macrophages (Figure 6C), and sites of sheath assembly, contraction, and disassembly colocalized with ClpB-fluorescence (Figure 6C), which is thought to mediate sheath disassembly in *Francisella* (Brodmann et al., 2017). While the T6SSs of *Vibrio* and *Pseudomonas* typically assemble all over the cells, the T6SS of *F. novicida* forms exclusively at the poles, a site that may be better suited to puncturing phagosomal membranes (Brodmann et al., 2017), whereas locations all over the cell are required for optimal interbacterial targeting as seen in videos of *Vibrio, Pseudomonas*, and *E. coli* (Basler and Mekalanos, 2012; Basler et al., 2013; Brunet et al., 2013). As there is no tape measure protein in T6SS classes 1–3, the upper limit to the length of the sheath is the width of the bacterium. Therefore, sheaths that form at the poles can extend the length of the bacterium (~1 micron for *F. tularensis*), whereas sheaths that form on the sides of the bacteria would be limited by the width of the bacteria (~ 0.5 micron). In the case of *V. cholerae*, the T6SS sheath contracts to 50% of its pre-contraction length (Wang et al., 2017); if the same holds true for *F. tularensis*, then a 0.8 micron sheath could penetrate 0.4 microns into the area of contact with the phagosomal membrane, whereas a sheath that formed at the side would have a shorter penetration distance.

## Individual components of the *Francisella* T6SS and their relation to components of other contractile secretion systems

### Structure and composition of the *Francisella* T6SS sheath

**Figure 7:**
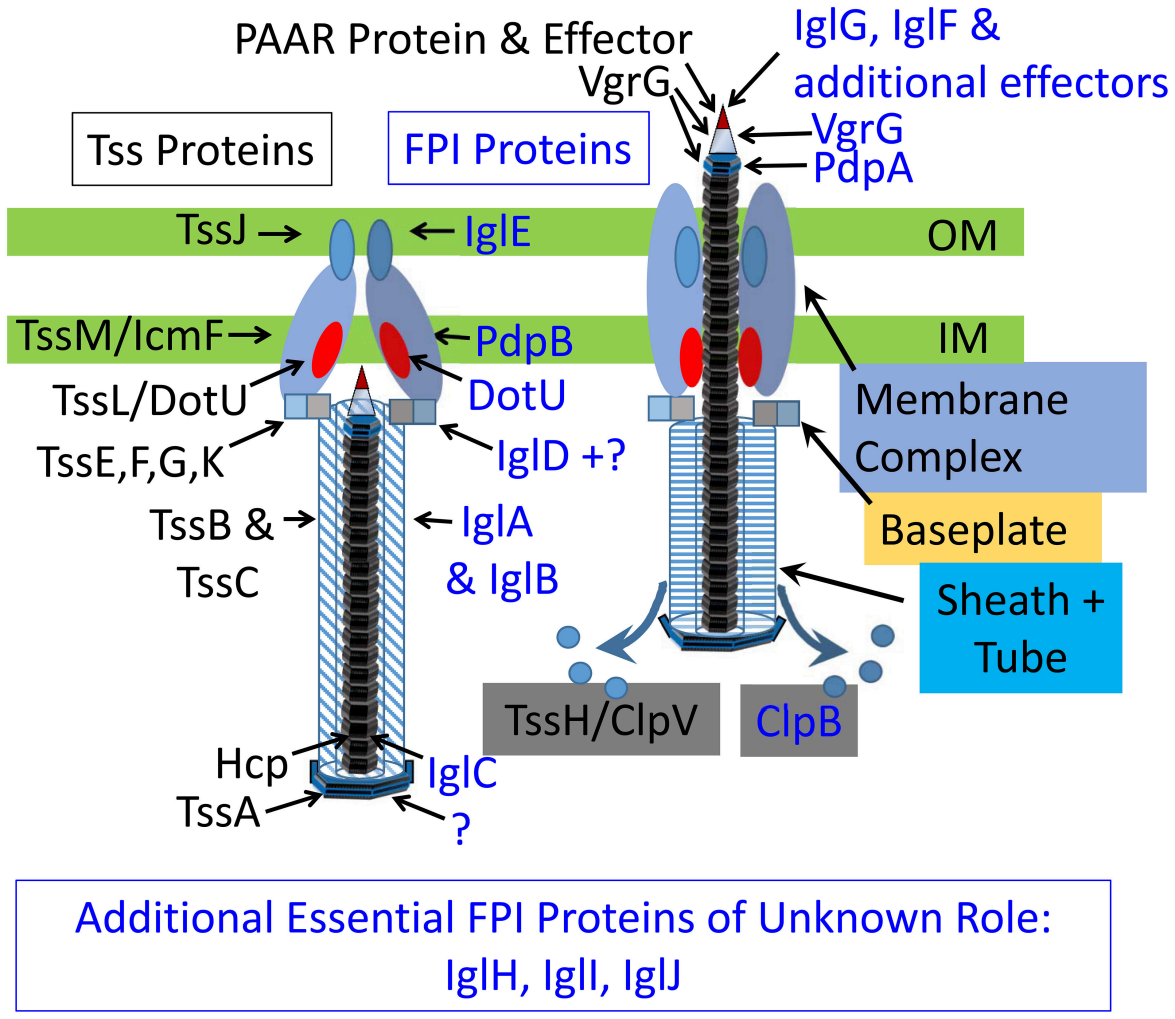
Schematic models of extended **(left)** and contracted **(right)** canonical and *Francisella* T6SS. Canonical T6SS subunits are labeled in black and *Francisella* T6SS subunits are labeled in blue.

The essential feature common to all CISs is a long sheath that contracts to propel the tube and central spike across a membrane (Figure 7). In the case of myophage and all eCISs reported thus far, including R-pyocins, Afps, and MACs, the contractile sheath is composed of a single protein. In contrast, in canonical T6SSs, the sheath protein is a heterodimer of TssB and TssC, with TssB corresponding to the N-terminus and TssC corresponding to the C-terminus of the T4 phage gp18 sheath protein. In *Francisella*, IglA and IglB have limited sequence homology with TssB and TssC, respectively, and were shown to co-immunoprecipitate by de Bruin et al. (2007). Bröms et al. demonstrated interaction between IglA and IglB in a yeast-2-hybrid system and identified a conserved α-helical region of IglA critical to the IglA-IglB interaction and to phagosome escape and intracellular replication (Bröms et al., 2009).

**Figure 8:**
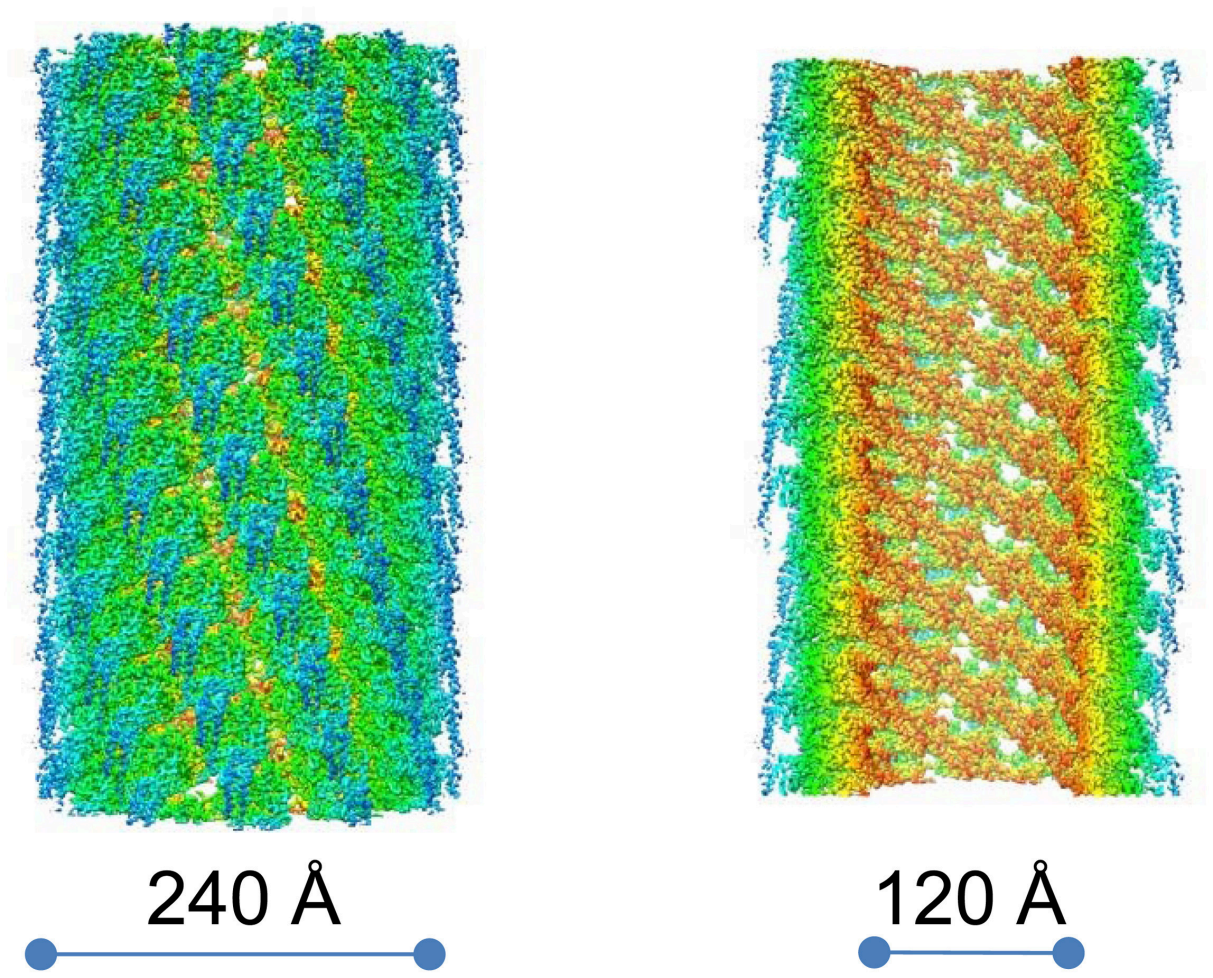
CryoEM density map of the contracted *F. novicida* T6SS sheath. Surface view **(left)** and cut-away view **(right)**. Prominent left-handed 6-start helices formed by surface ridges are apparent. Colored by radius, innermost colored brown and outermost colored blue. Reproduced with permission from (Clemens et al., 2015).

T6SS sheaths have been purified and atomic models have been prepared for *F. novicida* (Clemens et al., 2015) and *V. cholerae* (Kudryashev et al., 2015) in their contracted configurations and for a contraction-defective *V. cholerae* sheath in its extended conformation (Wang et al., 2017). In the case of *F. novicida*, we determined the structure of the contracted sheath at 3.7 Å resolution by cryoEM (Clemens et al., 2015; Figure 8). We showed that the asymmetric unit of the sheath, the IglA/IglB heterodimer, is an α-β-α sandwich, with the central β sheet of the sandwich formed by interdigitation of strands from both IglA and IglB. The IglA–IglB heterodimer shows remarkable structural homology with the gp18 and the R-pyocin sheath proteins despite only limited sequence homology (Clemens et al., 2015).

**Figure 9:**
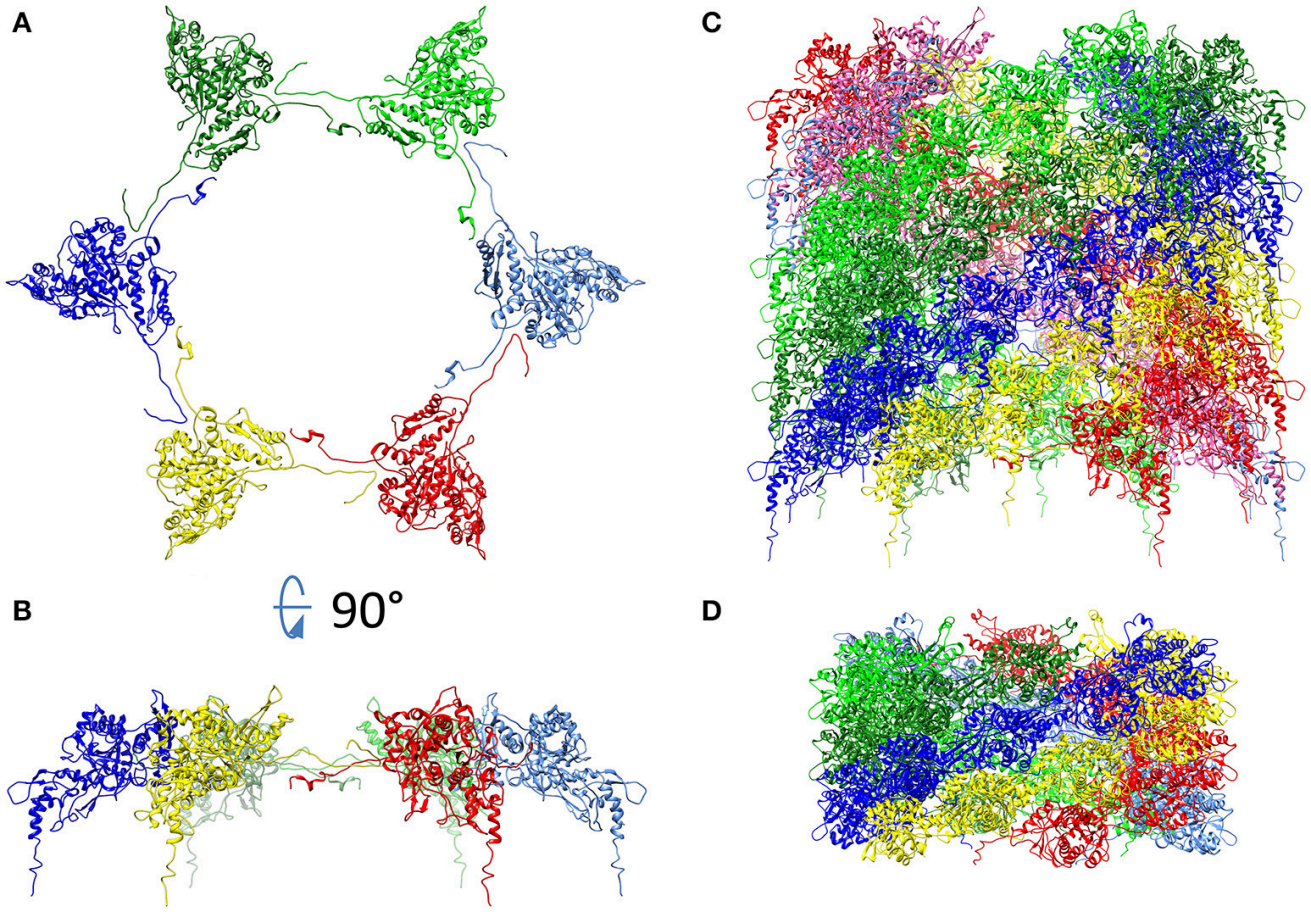
Hexagonal disc formed by six IglA/IglB heterodimers of *F. novicida* T6SS shown as viewed from the top **(A)**, from the side **(B)**, and stacked as a sheath of 11 discs **(C)**. For comparison, the contracted pyocin sheath (pdb 3J9R) is also shown **(D)**. Each heterodimer is shown as a ribbon diagram. In **(C)**, the IglA/B heterodimers of the bottom disc are colored as in **(B)** and the heterodimers that are connected by IglB β-strand connectors are given the same color. In **(D)**, pyocin sheath subunits connected by C-terminal β-strand connectors are given the same color. In both **(C)** and **(D)** the sheath subunits connected by β-strand connectors form 6-start right handed helices. **(A,B)** Adapted from Clemens et al. (2015) with permission.

We found that the contracted T6SS sheath consists of disks of 6 IglA/IglB heterodimers (Figure 9) that stack in a helical configuration (Clemens et al., 2015) and the contracted sheath shows a similar helical rise and turn to the contracted sheaths of T4 phage (Leiman et al., 2004), R-pyocin (Ge et al., 2015), and *V. cholerae* T6SS (Kudryashev et al., 2015).

**Figure 10:**
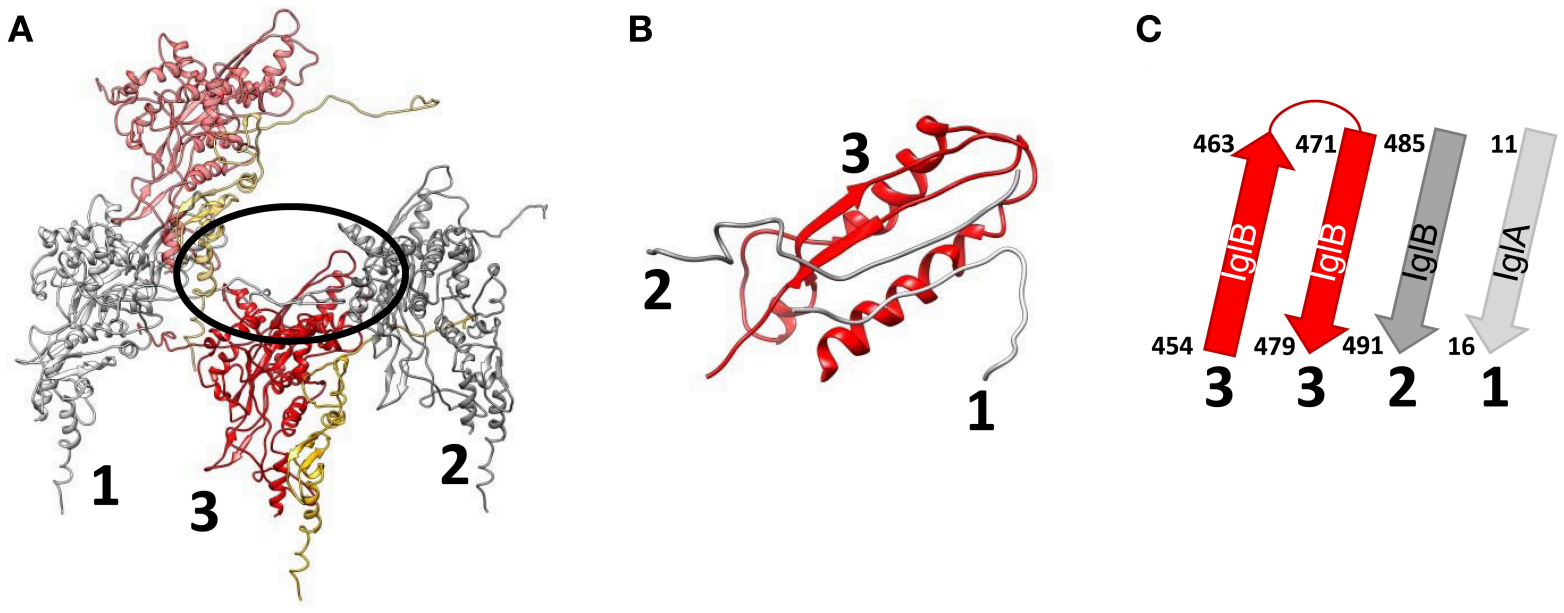
An extensively interwoven mesh of beta-strands links the subunits of the sheath. **(A)** Ribbon model of three interacting IglA/IglB dimers of a contracted sheath. IglA/IglB dimers from the same disc are labeled 1 and 2 and a dimer from the disc below is labeled 3. The area of their interaction through an augmented β sheet is circled. **(B)** Magnified view of the circled β sheet in **(A)**. **(C)** Ribbon diagram of the β sheet. Chains marked “1”, “2” and “3” in **(B,C)** come from the IglA/IglB dimers marked with the same number in **(A)**. Dimers #1 and #2, from the same disc, interact via the N-terminal and C-terminal β-strands of IglA and IglB, respectively, which augment a two-stranded anti-parallel β-sheet from near the C-terminus of IglB from dimer #3 of the disc below. Reproduced with permission from Clemens et al. (2015).

In the inner most layer of the contractile sheath of *F. novicida* (Clemens et al., 2015), an extensive interwoven meshwork of β-strands links the subunits of the sheath. Each disc of the sheath resembles the dancers in Matisse's “Dance,” with each of the 6 heterodimers of the disk holding hands with its adjacent partners on the disk. Specifically, the N-terminal beta strand of IglA interacts with the C-terminal beta-strand of IglB of the adjacent heterodimer. These two parallel β-strands augment a two-stranded anti-parallel β-sheet near the C-terminus of IglB from the disc below, i.e., the disc closer to the baseplate, (Figure 10). This interlacing of strands produces an extensively interwoven 2-dimensional meshwork which plays a dominant role in holding the subunits of the sheath together. A similar interwoven β-sheet meshwork has also been identified in the *V. cholerae* T6SS sheath (Kudryashev et al., 2015), and in R-pyocin (Ge et al., 2015).

Although the prominent surface ridges of the *Francisella* T6SS sheath form a left-handed 6-start helix (Clemens et al., 2015) which is of opposite handedness to the surface ridges of T4 phage, R-pyocin, and *V. cholerae*, the β-strand connections between IglA/IglB heterodimers of adjacent rings in the interwoven meshwork in the inner layer of the sheath form a right-handed 6-start helix (Figure 9C) in common with the sheaths of R-pyocin (Figure 9D), T4 phage, and *V. cholerae* T6SS and other T6SSs.

**Figure 11:**
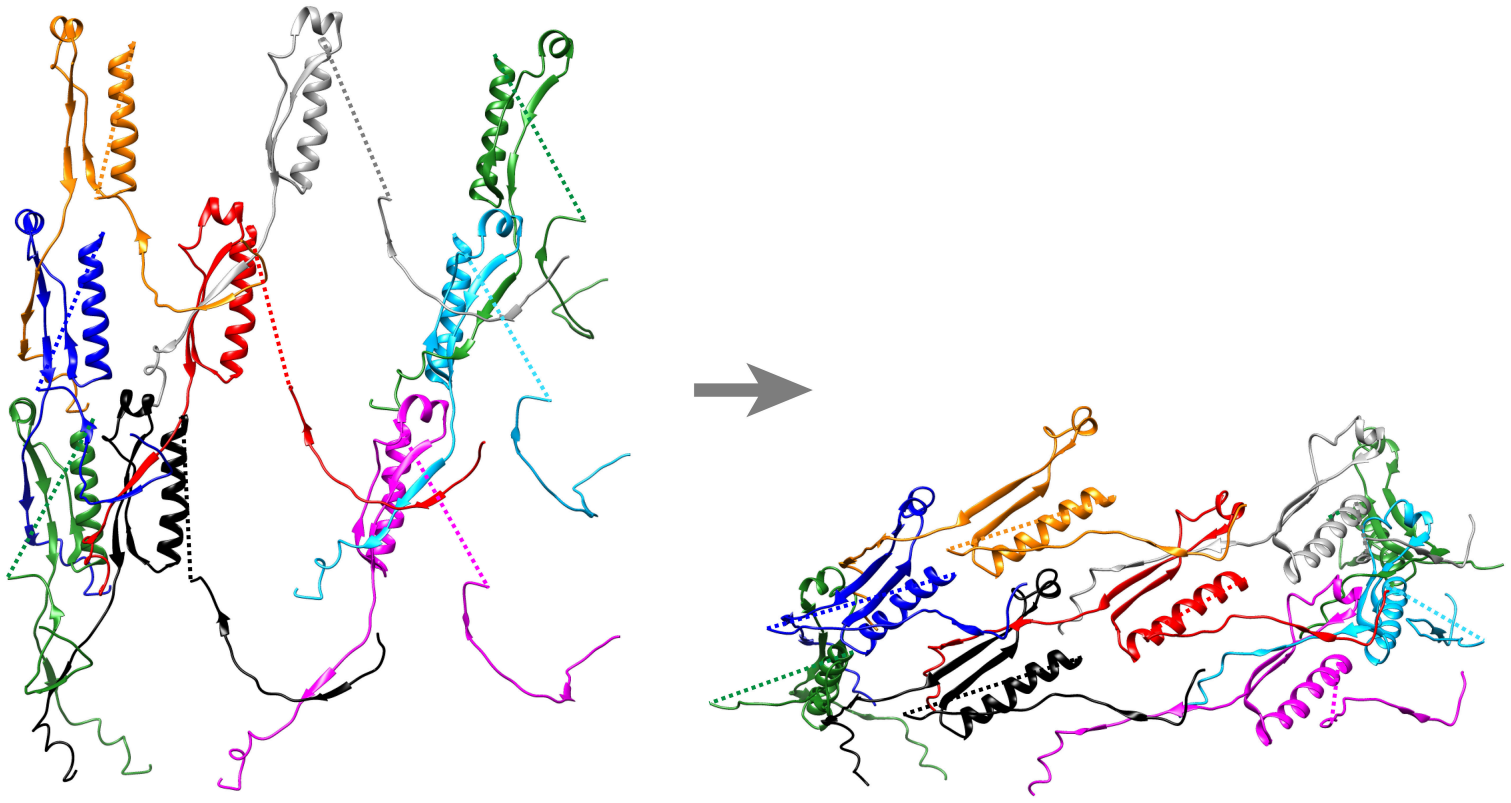
Ribbon diagram of pre- and post-contraction R-pyocin illustrating how the extensively interwoven β-strand meshwork holds the subunits of the contractile sheath together during contraction. Shown are the interconnecting β-strand arms and the tube attachment α-helices of 9 sheath subunits (3 subunits on each of three strands) of the extended **(left)** and contracted **(right)** atomic models of R-pyocin, viewed from outside the sheath with the baseplate at the bottom. To facilitate visualization of the interwoven mesh, the majority of each sheath subunit has been deleted and replaced by a dashed line connecting the N- and C-terminal segments of each subunit. Corresponding sheath subunits are given the same colors in the extended and contracted diagrams. Diagrams were created using Chimera (Pettersen et al., 2004) and the PDB atomic models of extended (3j9q) and contracted (3j9r) R-pyocin (Ge et al., 2015).

Ge and colleagues recently developed an atomic model of the conformations of pre- and post-contraction R-pyocin (Ge et al., 2015). The extended sheath is a metastable conformation that is stabilized by interactions with the inner tube and all of the energy required for contraction of the sheath is stored within the pre-contraction state (Ge et al., 2015). During contraction, the R-pyocin sheath subunits move largely as rigid bodies, rotating 85° on an axis almost perpendicular to the main axis of the sheath, causing the sheath to widen and contract. The subunits of the sheath are held together during this profound change in quaternary conformation by the extensively interwoven mesh of β-strands (Figure 11). The rigid-body rotation of the subunits, while still being held by the interlaced β-strand meshwork, occurs by virtue of hinge-like action at the N- and C-terminal arms of the sheath subunits (Ge et al., 2015). If Matisse had drawn a ring of dancers performing a sheath contraction, the dancers of the extended sheath would be standing, holding hands in a tight circle with their hands at their sides. In the contracted conformation, they would be lying on their right sides in a circle on the floor, still holding hands but now with their (N-terminal) right arms outstretched beyond their heads and their (C-terminal) left arms stretched beside their trunks. Thus, with contraction, the distance between partners in each ring increases, the diameter of the ring increases, and the rise between layers decreases. In the case of the canonical T6SS, contraction of the sheath rotates the sheath subunits outward, such that the most peripheral domain becomes accessible for disassembly by the ClpV-ATPase (Kudryashev et al., 2015; Wang et al., 2017).

We have shown that the interwoven β-strand meshwork is essential to function of the T6SS. Deletion of either the N-terminal β-strand of IglA or the C-terminal β-strand of IglB does not block formation of IglA-IglB heterodimers or formation of the fluorescent foci, but completely abolishes T6SS secretion and the capacity of the bacteria to escape their phagosome or to multiply intracellularly in macrophages (Clemens et al., 2015).

As hypothesized by Kudryashev et al. (2015) for the *V. cholerae* T6SS and shown by Taylor et al. (2016) in a pseudo-atomic model of T4 phage, the interwoven meshwork of the sheath continues into the baseplate, providing a strong link between the sheath and the baseplate. The C-terminus of the T4 sheath initiator protein, gp25, and its T6SS orthologue, TssE, both have three β-strands that resemble the C-terminal “handshake” domain of the sheath protein and are available to interact with the C-terminal β-strand of the sheath protein (gp18 or TssC, respectively) in the bottom disc of the sheath. Unfortunately, the corresponding baseplate protein in *Francisella* has not been identified. The β-strand meshwork has also been shown to be of critical importance for initiating and propagating sheath contraction. Basler's group has recently shown that insertion of 3–7 amino acids at residue 25, just after the VipA N-terminal (TssB) β-strand linker, blocks sheath contraction and allows the isolation of uncontracted sheaths with retained tube (Wang et al., 2017; Brackmann et al., 2018). CryoEM analysis of the non-contractile sheath revealed that the insertion led to altered connectivity between subunits, with the VipA N-terminal β-strand linker augmenting the 2 stranded anti-parallel β-strands of VipB of the adjacent heterodimer of the same disc, rather than of the disc below, as in the wild-type. The altered connectivity prevents contraction of the VipA-VipB mutant sheath because the VipA N-terminal β-strand is unable to stretch further to accommodate the conformational change required for sheath contraction. In their model, contraction is initiated by the baseplate pulling down on the N-terminal β-strand linkers of the first ring of the sheath, forcing a change in orientation of the subunits that propagates wave-like, ring-by-ring, from the baseplate to the last ring of the sheath (Wang et al., 2017; Brackmann et al., 2018).

## Secreted components of the T6SS

### The IglC tube

In all contractile injection systems, the contractile sheath wraps around a rigid central tube. In T4 phage, the tube is composed of gp19, which forms hexameric rings that stack to form the tube. The corresponding protein in canonical T6SS, Hcp, has sequence homology and structural homology to gp19. Interestingly, gp19 and Hcp show structural homology with the tube protein of non-contractile siphophage (Pell et al., 2009) (which lack a sheath), indicating an evolutionary linkage between contractile and non-contractile phage tail structures. In the case of myophage and siphophage, cargo of the phage tail delivery system (i.e., the tape measure protein and phage DNA) is injected into the target cell through the tube. For canonical T6SS, the effectors are associated (covalently or non-covalently) with VgrG at the tip of the tube rather than inside the tube. However, Hcp-associated effectors secreted inside the tube have also been demonstrated. For example, the *P. aeruginosa* **T**ype 6 **s**ecreted **e**ffector proteins 1–4 (Tse1-4) bind the interior of the Hcp-1 hexameric ring and point mutations in Hcp-1 that disrupt this binding block secretion of these Tse proteins without interfering with Hcp-1 or VgrG secretion, consistent with the secretion of these effectors via the interior of the Hcp-1 tube (Silverman et al., 2013; Whitney et al., 2014). Similar Hcp-associated effector proteins may exist in other bacterial T6SSs, though none have been identified in *Francisella*.

The atomic structure of Hcp of canonical T6SS has been determined (Mougous et al., 2006), as has the structure of the inner tube of R-pyocin (Ge et al., 2015) and T4 phage gp19 (Taylor et al., 2016). The tube protein monomer assembles into hexameric rings that stack to form the tube. The tube is thought to serve as a scaffold for assembly of the sheath and, accordingly, there is a one-to-one correspondence between sheath protein subunits and tube protein subunits. The atomic models of extended R-pyocin (Ge et al., 2015) and T4 phage tail (Taylor et al., 2016) show that the tube has the same helical rise and turn as the sheath. Consistent with a common evolutionary origin, the siphophage TP901-1 tube, R-pyocin tube, and T4 phage gp19 tube all have a similar helical turn and rise [22.4° and 38 Å for the siphophage TP901-1 tube (Bebeacua et al., 2013), 18.3° and 38.4 Å for R-pyocin tube Ge et al., 2015, and 17.9° and 40.2 Å for T4 phage gp19 tube Taylor et al., 2016, respectively]. CryoEM analysis of the extended *V. cholerae* sheath-tube complex with the VipA N-terminal insertion also showed that the Hcp tube had the same helical turn and rise as the sheath (Wang et al., 2017). While T6SS Hcp hexamers have been seen to stack in solution as tubes without any helical turn, it seems more likely that—as with its evolutionary relatives—it assembles within bacteria with the same twist as the sheath.

In the case of pre-contraction pyocin, an attachment α-helix near the C-terminus of the pyocin sheath protein interacts via reciprocally charged residues on the surface of the tube monomer (Ge et al., 2015). A similar interaction between an α-helix of TssC sheath units and Hcp was recently observed by cryoET analysis of the *Myxococcus xanthus* pre-contraction T6SS (Chang et al., 2017) and the T6SS of *V. cholerae*, both by CryoET subtomogram averaging of the wild-type pre-contraction sheath and at atomic resolution by cryoEM of a contraction-defective extended mutant sheath (Wang et al., 2017). In the case of *F. novicida*, an α-helix (residues 424–437) similarly situated on the interior of the sheath near the C-terminus of IglB has a region of charge complementary to corresponding residues on the surface of IglC (Clemens et al., 2015). Additional studies are required to determine whether these residues of IglB and IglC interact and stabilize the pre-contraction sheath conformation.

While there is homology between eCIS and T6SS tube proteins, there are also important differences. eCIS tubes remain together following secretion, such that it is common in micrographs of R-pyocins (Higerd et al., 1969; Govan, 1974), MACs (Shikuma et al., 2014), and Afp structures to see naked tubes or tubes that have been partially ejected (Govan, 1974; Shikuma et al., 2014). In contrast, T6SS tubes typically dissociate following secretion, suggesting that interactions with the sheath proteins are required to stabilize the interactions between the tube proteins.

The X-ray crystal structure of recombinant *Francisella* IglC purified from *E. coli* (Sun et al., 2007) was shown to have striking structural homology with canonical Hcp despite the absence of sequence homology (de Bruin et al., 2011). However, the X-ray crystal structure of the recombinant *Francisella* IglC shows an N-terminal 32 residue extension that would block assembly of the monomers into hexagonal discs (de Bruin et al., 2011). We have found by mass spectrometry that secreted IglC still bears its N-terminus (i.e., it is not proteolytically cleaved off prior to secretion), which raises the possibility that the X-ray crystal structure of recombinant IglC might differ from that of native IglC. The structures of the native IglC monomer and of the assembled IglC tube remain to be determined.

### The VgrG-PdpA central spike

In all CISs studied to date, the pre-contraction tube is tipped by a “central spike” protein complex that serves as a membrane-piercing needle. In T4 phage, the central spike complex is composed of two trimeric and one monomeric proteins: (gp27)_3_, (gp5)_3_, and (gp5.4)_1_. T4 phage gp27 is a trimer that forms the central hub of the baseplate, acting as an adapter between the 6-fold rotational symmetry of the baseplate and tube and the 3-fold symmetry of gp5. T4 gp5 is a highly intertwined trimer with a long beta-helix roll that is rich in valine and glycine. Its N-terminal oligonucleotide/oligosaccharide-binding (OB)-fold domain interacts with gp27 and its C-terminal apex domain interacts with the tip of the spike, gp5.4, a monomeric PAAR-motif containing protein (Taylor et al., 2016). In canonical T6SSs, a trimeric VgrG (valine-glycine repeat) protein is a functional fusion with both sequence and structural homologies to the gp27-hub and gp5-spike proteins, and in the case of “evolved VgrGs”, there are C-terminal extensions corresponding to the spike tip gp5.4. For example, VgrG1 of *V. cholerae* has a wide N-terminal head domain that corresponds to gp27 and the N-terminal OB domains of gp5, a beta-helix spike domain that corresponds to the beta helix spike of gp5, and a large, 513 amino acid C-terminal actin-cross-linking effector domain (Pukatzki et al., 2007). In other T6SSs, separate PAAR-repeat proteins are orthologues of T4 phage gp5.4 (Shneider et al., 2013) and bind to the C-terminus of the VgrG trimer, where they complete the membrane piercing tip and also recruit additional effector proteins (Shneider et al., 2013). Different PAAR-containing effector proteins can partner with the same VgrG protein, providing flexibility in the effectors that are secreted (Cianfanelli et al., 2016a). A remarkably diverse range of T6SS effector proteins have been identified that can intoxicate or kill the target cell by a variety of mechanisms. T6SS effector activities against bacteria include peptidoglycan hydrolases, phospholipases, pore forming proteins, and nucleases (Durand et al., 2014; Russell et al., 2014a), and those active against eukaryotic cells include cytoskeletal toxins [e.g., proteins causing ADP ribosylation of actin (Suarez et al., 2010) and actin cross-linking (Pukatzki et al., 2007)], and effectors that enhance uptake into epithelial cells (Sana et al., 2012), formation of multinucleated giant cells (Burtnick et al., 2011), inhibition of phagocytosis (Suarez et al., 2008), and red cell hemolysis (Böck et al., 2017).

*Francisella* VgrG protein has sequence homology with other VgrG proteins, but it is unusually short. Whereas, canonical VgrG proteins are typically 600–650 amino acids, *F. tularensis* VgrG is only 164 amino acid residues. Its short sequence and its appearance on EM negative staining suggest that it lacks both the N-terminal gp27-like head and any C-terminal effector extension. Indeed, bioinformatics modeling indicates that it even lacks the OB-fold of gp5. However, Eshraghi et al. have shown that PdpA is co-secreted with VgrG and co-immunoprecipitates with VgrG (Eshraghi et al., 2016). By TEM negative staining, PdpA resembles the cap on a VgrG needle (Eshraghi et al., 2016) and may functionally correspond to the gp27-like head domain and OB-fold domain of other VgrG proteins, providing an adaptor between the 3-fold symmetry of VgrG and the 6-fold symmetry of the tube and baseplate. In comparison with other VgrG proteins, the VgrG of *F. tularensis* and *F. novicida* is a truncated protein that lacks the N-terminal extensions of other VgrGs; PdpA may correspond to the missing N-terminal domains. Unlike canonical T6SSs, it is likely that the *Francisella* IglC tube and the baseplate proteins interact with PdpA rather than with the truncated VgrG.

### Additional secreted effector proteins

In addition to IglC, VgrG, and PdpA, several additional proteins have been shown to be secreted by the *Francisella* T6SS in liquid culture medium and in macrophages. Bröms et al. systematically expressed each of the 17 proteins of the FPI as fusion proteins in *F. tularensis* LVS with β-lactamase so that proteins secreted into the macrophage cytosol would be detected by cleavage of fluorescent substrate (Bröms et al., 2012). The authors detected fluorescent signal in macrophage cytosol with the β-lactamase fused to IglE, IglC, VgrG, IglI, PdpE, PdpA, IglJ, and IglF. Detection of a positive β-lactamase fluorescent signal was not observed when the fusion proteins were expressed in Δ*dotU*, Δ*vgrG*, Δ*iglC*, or Δ*iglG* LVS. Applying this method to *F. novicida* U112 required deletion of the FTN_1072 beta-lactamase gene. Fluorescent signal in the macrophage cytosol was observed with β-lactamase fused to IglE, IglC, PdpA, and PdpE, but not with VgrG, IglJ, IglF or IglI in the FTN_1072 deficient strain of *F. novicida*. The β-lactamase reporter is 29.5 kDa, which likely presents steric constraints and limitations on the proteins whose secretion can be detected by this assay system. As Nazarov et al. observed a 450 kDa cavity between the baseplate and the VgrG-PAAR protein complex of *V. cholerae* (Nazarov et al., 2018), there may be flexibility in the effector proteins that can be accommodated in assembly of the apparatus, and there may also be differences between *F. tularensis* LVS and *F. novicida* with regard to the effector proteins that are packed into this cavity. The observation of a positive fluorescence signal in the cytosol for IglE is intriguing, since IglE is generally thought to be an outer membrane lipoprotein corresponding to the membrane core complex protein, TssJ, rather than a secreted effector protein (Robertson et al., 2013; Nguyen et al., 2014). It is possible that IglE is released into the macrophage cytosol by a process of outer membrane blebbing when the *Francisella* replicate extensively in the host cell cytosol.

Rigard et al. used high KCl to induce T6SS secretion by *F. novicida* in liquid culture medium and demonstrated T6SS-dependent secretion of IglF and IglG (Rigard et al., 2016). Based on *in silico* analysis, they proposed that IglG is a PAAR-like protein that recruits IglF to the VgrG spike (Rigard et al., 2016). Eshraghi et al. compared proteins secreted by wild type and Δ*dotU F. novicida* in broth culture containing high KCl to identify T6SS proteins secreted in a T6SS-dependent fashion by mass spectrometry based proteomics. Their analysis identified five FPI encoded proteins—IglC, VgrG, PdpA, PdpC, and PdpD—and several proteins encoded on genes outside of the FPI (labeled OPI for “outside pathogenicity island”) as being secreted by the T6SS—OpiA, OpiB-1, and OpiB-3. Whereas, IglC, VgrG, and PdpA are all interdependent for secretion by the T6SS, disruption of genes encoding PdpC, PdpD, OpiA, or OpiB did not block secretion of the other proteins. Because *pdpC, pdpD*, and the *opi* genes can be disrupted without impacting secretion, they are presumed to encode effector functions. However, their actual biological functions are not known. While disruption of *iglC, pdpA*, or *vgrG* abolishes *F. novicida* growth in macrophages and virulence in animals, disruption of *pdpC* or *pdpD* has an intermediate effect, as these genes are required for virulence in animals, but they are not essential for intracellular growth in macrophages (Ludu et al., 2008; Long et al., 2013). In the highly virulent *F. tularensis* SCHU S4 strain, disruption of both copies of *pdpC* caused a delay in phagosome escape and a modest decrease in intracellular growth in J774 mouse macrophages (Long et al., 2013). Following intranasal challenge, the *pdpC* double deletion mutant was able to disseminate to liver and spleen, but did not cause death and was ultimately cleared by mice (Long et al., 2013). Similarly, Uda et al. studied a SCHU strain of *F. tularensis* which has been attenuated by serial passage on artificial media (Uda et al., 2014). They restored full virulence by serial passage (9 passages) in mice and found that the only difference between the original attenuated strain and the virulent strain (“P9”) was a single nucleotide difference in one of the two copies of *pdpC*, such that the original strain expressed only truncated PdpC whereas the P9 strain with restored virulence expressed both a truncated and a full length PdpC (Uda et al., 2014). Uda et al. confirmed the importance of PdpC by disrupting both copies of *pdpC* in the P9 strain and showing that the Δ*pdpC* strain had reduced growth in mouse J774.1 macrophages and reduced virulence in mice, and that both attenuations were complemented with intact *pdpC*. Brodmann et al. recently demonstrated that *pdpC* and *pdpD* are not required for T6SS assembly in *F. novicida*, whereas *iglF, iglG, iglI*, and *iglJ* are required (Brodmann et al., 2017). Disruption of *pdpC* or *pdpD* genes markedly impaired phagosome escape and intracellular growth, but the defect was less severe than the defect resulting from disruption of *iglF, iglG, iglI*, or *iglJ* (Brodmann et al., 2017). Disruption of either *pdpC* or *pdpD* also impaired *F. novicida* virulence in mice, but the defect was less severe than disruption of the gene encoding the membrane complex protein, *pdpB* (Brodmann et al., 2017). On the other hand, disruption of both *pdpC* and *pdpD* resulted in *F. novicida* that were completely unable to escape their phagosome or cause disease in mice, i.e., defects as profound as that caused by Δ*pdpB* (Brodmann et al., 2017). In contrast, disruption of *pdpE* has no impact on phagosome escape in macrophages or virulence in mice in either *F. novicida* (Brodmann et al., 2017) or *F. tularensis* LVS (Bröms et al., 2011). Eshraghi et al. (2016) observed that disruption of *pdpD* or any of the *opi* genes, individually, had little or no impact on the capacity of *F. novicida* to multiply in macrophages. However, combined deletion of *pdpC, pdpD, opiA*, and *opiB* seriously impaired intracellular growth without impacting core T6SS secretion function. None of these genes are found in species outside of *Francisella* and their function remains unknown (Eshraghi et al., 2016). It is tempting to speculate that the effectors that are required for phagosome escape and intracellular growth include pore forming and phospholipase activities that could lead to formation of the characteristic fibrillar coat and phagosome escape.

Assuming that PdpA-VgrG-IglG correspond to the VgrG-PAAR protein complex of canonical T6SS, then IglF, PdpC, PdpD, OpiA, OpiB, PdpE, and possibly IglI may be additional effectors packaged within a ~450 kDa sized baseplate cavity similar to that described for *V. cholerae* (Nazarov et al., 2018). As the total mass of these monomeric proteins is 491–577 kDa (depending on which OpiA or OpiB proteins are packaged and whether or not IglI is included), it is possible that some, but not all, are packaged within an individual T6SS apparatus. *Francisella* bacteria may preferentially package some effectors as opposed to others depending on environmental stimuli. Small effectors could fit within the IglC tube and almost all Hcp-associated effectors in other T6SSs are <20 kDa, which—for globular proteins—is a size that can fit within the 40 Å cavity of the Hcp tube (Whitney et al., 2014). However, other than VgrG and the presumed PAAR-like protein, IglG, none of the putative effectors listed in Table 3 are <20 kDa. PdpE is 21.6 kDa, but attaching the 29.5 kDa β-lactamase fusion protein (Bröms et al., 2012), which is 43 Å in its smallest dimension, would sterically prevent it from fitting within the tube. Thus, the secreted β-lactamase signals for proteins, such as PdpE, observed by Bröms et al. (2012), would require packaging in the cavity between VgrG and the baseplate, rather than inside the tube. On the other hand, proteins for which Bröms et al. (2012) did not see a signal could conceivably fit within the tube in an unfolded or linear conformation, in a fashion analogous to the tape measure protein.

**Table 3:** Size of putative *Francisella* T6SS secreted proteins.

**Putative effector proteins**	**Size (kDa)**
VgrG	17.5
PdpA	95.3
IglG	18.3
IglF	67.9
IglI	44.6
PdpC	155.6
PdpD	140.7
PdpE	21.6
OpiA (FTN_0131)	50.7
OpiA-1 (FTN_1069)	91.2
OpiB-1 (FTN_1071)	54.9

### Baseplate components

**Figure 12:**
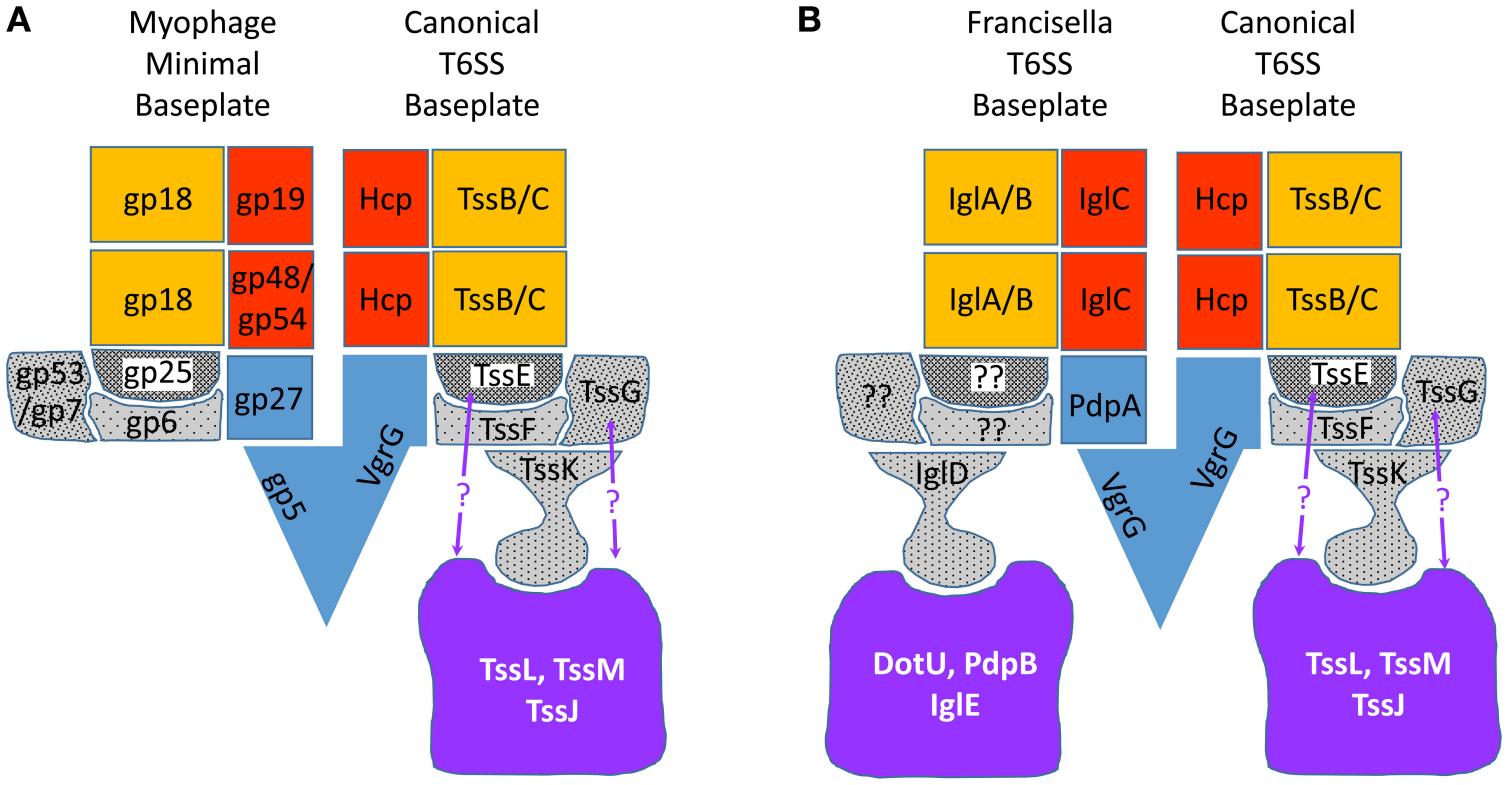
Comparison of models of myophage and T6SS baseplates. **(A)** Myophage minimal baseplate structure (left side of the model, modified from Leiman and Shneider (2012) and the canonical T6SS baseplate (right side of the model). **(B)** Proposed *Francisella* baseplate (left side of model) compared with canonical T6SS baseplate (right side of model). Baseplate wedge proteins are shown in gray with stippling, central hub/spike proteins in blue, sheath proteins in gold, tube proteins in red, and membrane complex in lavender. Interactions between TssE and TssG and the membrane complex (indicated with purple question marks) are suggested by B2H and immunoprecipitation studies (Brunet et al., 2015; Logger et al., 2016; Zoued et al., 2016a) of EAEC baseplate proteins.

In all CISs, a baseplate serves as a platform for assembly of the tube and sheath. In T6SS, the baseplate has the additional function of anchoring the sheath to the membrane complex. In T4 phage, the baseplate is a highly complex structure made up of 145 polypeptide chains of 15 different proteins that assemble as six wedges around a central hub (Taylor et al., 2016). Other contractile phage, such as P2, have much less complex baseplate structures with only four different proteins: gpV (homologous to hub/spike proteins gp27and gp5) and wedge components W (gp25-like sheath initiator), gpJ (gp6-like sheath platform) and gpI (gp53/gp7-like linker). This prompted Leiman and Shneider to propose the concept of a minimal tail tube structure with a simplified baseplate consisting of a central hub and three wedge proteins orthologous to gp6, gp25, and gp53/gp7 (Leiman and Shneider, 2012; Figure 12). Using the T4 baseplate nomenclature, the central hub of the minimal baseplate consists of the gp5-like spike and the gp27-like component that acts as an adaptor between the 3-fold symmetry of the gp5 spike and the 6-fold symmetry of the tail tube. Gp6, gp25, and gp53/gp7 proteins form a wedge and six wedges assemble to form a hexagonal baseplate that embraces the central hub. Gp25 is at the center of each wedge and recruits sheath protein gp18 to initiate the polymerization of the sheath. Gp6 is a central component of each wedge, holding the wedges together and interacting with gp25 (at the center) and gp53/gp7 (at the periphery) of each wedge. Gp53/gp7, at the periphery of the wedge, interacts with tail fiber receptors and with gp6 (Leiman and Shneider, 2012).

Because the T6SS baseplate must fulfill the same functions as the phage baseplate, it is anticipated that it will show a structure similar to the myophage minimal baseplate, but with additional features that anchor it to the membrane complex (Figure 12A). As described above, VgrG of canonical T6SS is homologous to gp5 and gp27 and forms the hub of the baseplate. TssE shares homology with T4 baseplate protein gp25, the sheath initiator protein, but the assignment of other proteins to the T6SS baseplate are less clear. Brunet et al. developed Hcp Cys-substitution mutants of enteroaggregative *Escherichia coli* (EAEC) as a biochemical tool to report proper head-to-tail stacking of Hcp hexameric rings vs. improper tail-tail or head-head hexameric stacking, as assessed by SDS-PAGE and Western immunoblotting after *in vivo* oxidative cross-linking (Brunet et al., 2014). Since a functional baseplate is required as a platform for assembly of the tube of tailed bacteriophages, Brunet et al. reasoned that proper Hcp assembly could serve as a surrogate for functional T6SS baseplate assembly. Consistent with this hypothesis, they found that strains in which the baseplate hub VgrG was deleted, formed aberrant head-to-head and tail-to-tail Hcp interactions, whereas the parental EAEC formed only the correct head-to-tail Hcp multimers (Brunet et al., 2014). Interestingly, the sheath proteins TssB and TssC were not required for formation of the correct Hcp interactions. Using this assay, Brunet et al. demonstrated that 6 proteins—TssA, TssE, TssF, TssG, TssK, and VgrG—were required for formation of the correct Hcp interactions, suggesting that these 6 proteins are required for functional baseplate assembly (Brunet et al., 2015). Bioinformatic analysis showed secondary structural similarities between part of TssF and the P2 phage baseplate protein J and the corresponding T4 phage baseplate wedge protein gp6. Secondary structure similarities were also apparent between TssG and the P2 phage baseplate protein I (Brunet et al., 2015), which may correspond to T4 phage baseplate wedge protein gp53 or gp7 (Leiman and Shneider, 2012). Brunet et al. used bacterial-2-hybrid (B2H) analysis to show that TssF interacts with TssG and Hcp, and that TssG interacts with Hcp, TssC, TssE, and TssF. Immunoprecipitation pull-down studies of heterologously expressed protein lysates confirmed these interactions and also showed that TssF and TssG co-immunoprecipitated with TssE, and that VgrG pulled down TssF and TssG, and also pulled down TssE, -F, and -G (Brunet et al., 2015). In B2H analysis, TssK gave a positive signal when co-expressed with TssG and TssF, but not when expressed with either TssG or TssF alone, indicating the TssK interacts with the TssF-G complex. Genetic mutational analysis and fluorescence microscopy studies showed that EAEC expressing sfGFP-TssF or sfGFP-TssK formed discrete fluorescent foci near the inner membrane of both wild-type and Δ*tssBC* EAEC strains, consistent with the idea that the baseplate assembles on the membrane prior to sheath assembly. Indeed, in EAEC expressing both sfGFP-TssF or sfGFP-TssK and mCherry-TssB, sheath elongation occurred after and at the site of the GFP-TssF or TssK focal fluorescence. In Δ*tssK* strains, sfGFP-TssF fluorescence was diffuse and no fluorescent foci formed, indicating that TssK is required for baseplate assembly. In Δ*tssM* EAEC strains, the sfGFP-TssF fluorescence was mostly diffuse, but some foci continued to form; these moved freely around the cytosol rather than appearing anchored to the membrane, consistent with the idea that the baseplate is anchored to the TssM-containing membrane complex (Brunet et al., 2015). Conversely, fluorescent foci of GFP-TssM form at the membrane even in Δ*tssK* and Δ*tssF* strains, consistent with the idea that membrane core complex formation precedes baseplate assembly. Since TssK interacts with cytoplasmic loops of TssL and TssM (Zoued et al., 2013), the data suggest a model in which the membrane complex forms first and recruits TssK, which in turn recruits the additional baseplate proteins TssF, -G, and -E (Brunet et al., 2015). Consistent with this model, Taylor et al. (2016) have recombinantly expressed TssE, -F, -G, and -K proteins and shown that they form a stable complex with an apparent stoichiometry of TssE_1_F_2_G_1_K_3._

Since the baseplate connects the sheath to the membrane complex, it must form interactions with both, and these interactions have been examined by B2H analyses and immunoprecipitation studies. As noted above, TssE is thought to serve as a gp25-like sheath initiator and to anchor the sheath to the baseplate through an extension of the interwoven β-strand meshwork. B2H analyses and immunoprecipitation studies of EAEC T6SS have shown that TssE interacts with the cytosolic domain of TssL (Zoued et al., 2016a), suggesting that TssE interacts with the sheath, VgrG, and the membrane core complex. B2H and immunoprecipitation studies have shown that TssG interacts with the cytoplasmic loop of the inner membrane protein TssM (Brunet et al., 2015; Logger et al., 2016), and that TssK interacts with both TssM and TssL (Zoued et al., 2013; Brunet et al., 2015; Logger et al., 2016; Nguyen et al., 2017).

The X-ray crystal structure of TssK shows a trimeric protein with an hourglass shape and three domains: a broad N-terminal shoulder, a thin neck, and a broad C-terminal head (Nguyen et al., 2017). The N-terminal shoulder domain is structurally and functionally similar to siphophage receptor binding protein (RBP) shoulder domains (Nguyen et al., 2017). Myophage such as T4 and P2 have no protein with sequence or structural homology with TssK. It is intriguing that, in evolution, T6SS has borrowed a structure from siphophage to provide a link between the baseplate and the membrane complex, a structure absent from phage. B2H studies show that the N-terminal shoulder domain interacts with the rest of the baseplate and that the C-terminal head interacts with the membrane complex. The connection between the C-terminal head domain and the neck is very flexible and this flexibility is hypothesized to allow TssK to maintain a stable link between the baseplate and the membrane complex during conformational changes accompanying contraction (Nguyen et al., 2017). In addition to linking the baseplate to the membrane complex, it has been reported that TssK interacts with TssC in Enteroaggregative *E. coli* (EAEC) (Zoued et al., 2013), suggesting that it may also help link the sheath to the baseplate. However, cryoET of the *M. xanthus* T6SS shows a distance of 300 Å between the sheath and the membrane complex (Chang et al., 2017), which is more than twice the 110 Å length of TssK (Nguyen et al., 2017). While it might be envisioned that TssK oligomerization could bridge this distance, in siphophage the RBP shoulder domains all lie in the same plane of the baseplate, perpendicular to the main axis of the sheath (Legrand et al., 2016).

T6SS baseplate structures have been observed at low resolution by cryoET of intact *M. xanthus* (Chang et al., 2017) and at a finer 8.0 Å resolution by cryoEM of isolated sheath-baseplate complexes from contraction-defective VipA-N3 *V. cholerae* (Nazarov et al., 2018), but the resolution has not been sufficient to assign proteins definitively to the observed electron densities in the baseplate. Nazarov and colleagues observed a central spike surrounded by six structures similar to the phage baseplate wedges, but with an additional density hanging down from each wedge in a position suited to connect to the membrane core complex. They were able to fit the X-ray crystal structure of VgrG_3_ and PAAR monomer to their central spike electron density, and use the protein density volume-to-mass coefficient to estimate a combined mass for the proteins in the baseplate wedge of 191.2 kDa, which is a good match to the calculated mass of 189.2 kDa, based on amino acid sequence, for a wedge complex consisting of TssE_1_F_2_G_1_ (Nazarov et al., 2018). Nazarov et al. determined the structure of the “connector protein” densities hanging down from each wedge to a resolution of 10 Å and observed a good fit with the X-ray crystal structure of the shoulder and neck domains of EAEC TssK trimer determined by Nguyen et al. The head domain was not resolved, presumably because of its flexibility. Nazarov et al. observed that the putative TssK_3_ “connector proteins” are situated in the T6SS baseplate in a position corresponding to that of domain IV of the trimeric gp10 proteins at the periphery of the T4 phage baseplate. Whereas, domain IV of gp10_3_ connects gp7 in the T4 phage baseplate to the tail fiber network, TssK_3_ in the T6SS baseplate would connect TssG to the membrane complex (Nazarov et al., 2018). In their model, the VgrG/PAAR central spike complex is surrounded by a spacious cavity that can accommodate up to ~450 kDa of effector proteins (Nazarov et al., 2018). This model is consistent with TssK interactions with baseplate proteins, TssF and TssG, and with membrane complex proteins, TssL and TssM, but not with TssK interactions with the sheath (Figure 12). In addition, while the model is consistent with TssE interacting with both the sheath and with TssF and TssG, it is difficult to visualize interactions between the membrane complex and TssE or TssG (Figure 12). While it is possible that this could reflect differences between baseplates of EAEC and *V. cholerae*, it is also possible that some protein-protein interactions observed in B2H systems and pull-down studies do not reflect interactions in an assembled sheath. After all, Hcp can form aberrant head-to-head and tail-to-tail interactions that do not reflect its interactions in an assembled T6SS.

#### The Francisella T6SS baseplate

While IglD shows extremely limited homology with TssK and is assumed to be part of the baseplate, the FPI proteins that correspond to TssE, TssF, and TssG remain to be determined and the structure of the *Francisella* T6SS baseplate is unknown (Figure 12B). It is noteworthy that the spacious cavity surrounding the *V. cholerae* T6SS central spike could accommodate multiple effector proteins identified by Eshraghi et al. (2016).

### Membrane complex

Canonical T6SSs have a membrane complex that creates a channel spanning the inner and outer membranes. The membrane complex functions to anchor the T6SS to the membrane and allows secretion to occur without loss of membrane integrity (Ho et al., 2014). The membrane complex is a key feature that differentiates T6SSs from other CISs, which, functioning extracellularly, have no need for such a structure. In the case of canonical T6SS, two integral inner membrane proteins—TssM and TssL—and one outer membrane lipoprotein, TssJ, have been identified as components of the membrane complex (Ma et al., 2009; Felisberto-Rodrigues et al., 2011; Durand et al., 2012). TssL and TssM have homology with the inner membrane proteins, DotU and IcmF, respectively, of the Type 4 Secretion System (Ma et al., 2009; Durand et al., 2012), suggesting that these components were inherited from the T4SS during evolution of the T6SS.

The X-ray crystal structures of some of the soluble cytosolic domains of the membrane complex have been determined. In addition, the TssJLM proteins of EAEC have been co-expressed as an epitope-tagged protein complex in *E. coli* BL21, allowing affinity purification of the recombinant TssJLM complex and determination of its structure at 11 Å resolution by TEM analysis of negatively stained particles (Durand et al., 2015). The 1.7 MDa particles exhibited 5-fold symmetry and consisted of 30 polypeptide chains, with 5 dimers of TssJLM heterotrimers. The integral inner membrane protein TssM interacts with TssJ via its C-terminal periplasmic domain, and it interacts with TssL via its cytoplasmic domain (Zheng and Leung, 2007; Ma et al., 2009, 2012; Felisberto-Rodrigues et al., 2011; Rao et al., 2011; Durand et al., 2015). Structural analysis and chemical modification studies with a membrane impermeant reagent are consistent with a model in which the complex undergoes a conformational change during secretion, with the tip of the complex opening akin to the way the leaves of a camera shutter move to form an aperture, thereby allowing passage of the tube and spike complex (Durand et al., 2015).

Stable interaction between a membrane complex with 5-fold symmetry and a baseplate with 6-fold symmetry is problematic. The attachments between sheath, baseplate, and membrane complex must have a strength comparable to that conferred by the interwoven meshwork (“handshakes”) that holds the sheath together during contraction (Kudryashev et al., 2015). However, as the TssJLM proteins were overexpressed in the absence of other components of the T6SS, it is possible that the symmetry of the purified complex differs from that of the *in situ* structure. Noting that TssA interacts with TssJM prior to recruitment of TssL, Cascales group has suggested that the star-shaped, dodecameric TssA acts as a chaperone that imposes a 6-fold symmetry onto the TssJLM membrane complex as it forms (Zoued et al., 2017).

#### The Francisella T6SS membrane complex

Based on limited sequence homologies, mutational analysis, immunoprecipitation and B2H analyses, IglE, DotU, and PdpB have been proposed as orthologues of TssJ, TssL, and TssM, respectively (Robertson et al., 2013; Nguyen et al., 2014). Although *F. novicida* DotU shows only 15% identity to TssL, X-ray crystallography of the *F. novicida* DotU soluble domain (Robb et al., 2012) shows structural homology to the soluble TssL domain of EAEC (Durand et al., 2012). PdpB shows only 18% identity to TssM/IcmF of *V. cholerae*, but appears functionally to resemble TssM/IcmF because it is an inner membrane protein that interacts both with DotU and with the outer membrane lipoprotein, IglE. Although IglE has no sequence homology with TssJ, it undergoes palmitoylation at a cysteine residue that anchors it to the outer membrane and replacement of this cysteine with a glycine abolishes both palmitoylation and multiplication of the bacteria in macrophages (Nguyen et al., 2014). Consistent with IglE being the orthologue of TssJ, B2H screening and immunoprecipitation studies demonstrate that IglE interacts with the periplasmic C-terminus of PdpB (Nguyen et al., 2014).

While there is good evidence that IglE, DotU, and PdpB contribute to the membrane complex of *Francisella*, there may be additional proteins within or outside of the FPI that contribute to the membrane complex. For example, in some cases it has been shown that the T6SS membrane complex is inserted and anchored into the membrane in concert with additional proteins that have peptidoglycan binding and degrading activities (Aschtgen et al., 2010; Weber et al., 2016; Santin and Cascales, 2017). As *Francisella* has an extensive capsule that could present a barrier to T6SS operation, it is tempting to speculate that similar peptidoglycan and capsule binding and degrading proteins might be recruited to the *Francisella* membrane complex.

### FPI proteins of unknown function

Several proteins of the FPI are essential for intracellular growth and virulence of *Francisella* in animals, yet their specific role has not been determined. These proteins may correspond to key components of canonical T6SS, such as the chaperone protein TssA, or baseplate proteins TssE, TssF, and TssG, whose corresponding proteins in *Francisella* have not yet been identified.

## Non-FPI components

### ClpV

An essential component present in all canonical T6SSs is a ClpV ATPase that functions to disassemble the contracted sheath and enable dynamic recycling for repeated rounds of firing, disassembly and reassembly. Myophage, R-pyocins, Afps, and MACs have no requirement for a gene corresponding to ClpV because their contractile apparatus contracts once and is not recycled. The *Francisella* T6SS is an outlier in that none of the genes within the FPI gene cluster encode a ClpV ATPase. However, Brodmann *et al*. recently demonstrated that *F. novicida* employs ClpB ATPase, encoded outside of the FPI, to disassemble its contracted T6SS sheath (Brodmann et al., 2017). In live fluorescence imaging, ClpB-mCherry fluorescence colocalizes with sites of IglA-sfGFP sheath assembly, contraction, and disassembly, as shown in Figure 6C (Brodmann et al., 2017). Canonical T6SSs have a conserved sequence on their sheath protein, an α-helical region at the N-terminus of VipB which includes a consensus sequence “LLDEIM” (residues 19–24 of the *V. cholerae* TssC homolog, VipB) that is bound by the ClpV ATPase (Pietrosiuk et al., 2011). However, *F. tularensis* IglB has no similar α-helical region or consensus sequence. The sheath sequence recognized by ClpB ATPase has not been determined, but presumably is more exposed and accessible to binding in the contracted than in the pre-contracted sheath conformation.

### Antennae

Contractile injection systems are thought to function in a contact dependent fashion, with sheath contraction occurring upon interaction between the system and the target surface. In the case of myophage, receptor binding proteins are connected via tail fibers to the baseplate (Bartual et al., 2010). R-pyocins (Higerd et al., 1969), MACs (Shikuma et al., 2014), and Afps (Heymann et al., 2013) have been shown to have similar tail-fiber structures connecting to their baseplates. It is thought that interaction between the tail fiber receptor and its ligand triggers conformational changes in the baseplate that in turn lead to opening of the baseplate and sheath contraction. For T6SS, ECT of intact *M. xanthus* bacteria has visualized tail fiber-like antennae with terminal bulbs suggestive of receptor binding proteins (Chang et al., 2017). The genes encoding these structures are not known and most likely reside outside of the T6SS gene cluster as no genes homologous to tail fiber proteins are present within the *M. xanthus* T6SS gene cluster (Chang et al., 2017). In addition, as connections between the antennae and the T6SS baseplate or membrane complex have not been visualized, it is unclear whether the observed antennae are truly T6SS elements.

Because *F. tularensis* T6SS functions to mediate phagosome escape, it is tempting to speculate that it possesses similar antennae-like sensors that interact with molecules within the host phagosome or on the phagosomal membrane to trigger contraction.

### The T6SS assembly process

The sequence of events involved in assembly of canonical T6SS has been dissected by genetic mutational analysis and fluorescence studies in EAEC (Brunet et al., 2015; Zoued et al., 2016b). These studies have revealed that assembly of the T6SS begins with recruitment of TssM to TssJ. TssA, acting as a chaperone, then binds to the TssMJ prior to recruitment of TssL and formation of the membrane complex. As noted above, the TssA complex, with its 6-fold symmetry, could impose a similar symmetry during assembly of the TssJLM membrane complex (Zoued et al., 2017). The baseplate then assembles at the site of the membrane complex and polymerization of the sheath and tube follows (Brunet et al., 2015), again with a requirement for TssA acting as a chaperone to ensure proper assembly, though TssA does not become a part of the final assembled baseplate (Zoued et al., 2017). Polymerization of the sheath and tube then occur, and it has been shown that TssA is required for proper stacking of Hcp hexamers and for extension of the sheath, leading to the hypothesis that the star shaped TssA complex acts as a chaperone to ensure proper assembly of each new hexagonal layer of the tube and extended sheath (Zoued et al., 2017). As the tube and sheath assemble, the TssA complex remains associated with the distal end of the sheath (Zoued et al., 2016b). In the final extended sheath, TssA may function in a fashion analogous to the T4 phage gp3/gp15 tube/sheath terminator proteins, serving to stabilize the extended sheath and ensure proper expulsion of the tube during contraction (Zoued et al., 2016b). CryoEM of extended sheaths from the contraction-defective VipA-N3 expressing strain of *V. cholerae* by Basler's group revealed cap-like structures at the distal end of the sheaths (resolved at 7.5 Å) with a star-like configuration and an estimated mass of 540 kDa, consistent with either dodecameric TssA or an unusual configuration of the terminal VipA/B sheath subunits (Nazarov et al., 2018). An alternative model has been proposed by Planamente et al., who also observed dodecameric rings of *Pseudomonas* TssA1 at the ends of T6SS sheaths, demonstrated interaction of TssA1 with TssK1 and TssF1 by pull-down experiments, and noted sequence similarity between the TssA1 and the C-terminus of gp6, leading them to propose a gp6-like role for the *Pseudomonas* TssA1 in the baseplate (Planamente et al., 2016). However, TssA of EAEC lacks the gp6-like domain found in *Pseudomonas* TssA1, and whereas TssA is essential to EAEC T6SS assembly and function, TssA1 is not essential to *Pseudomonas* T6SS function, suggesting that EAEC TssA and *Pseudomonas* TssA1 have different structures and functions (Zoued et al., 2017).

While it is likely that the *Francisella* T6SS is assembled in a similar fashion, the *Francisella* orthologue to TssA of EAEC has not been identified.

## Summary

Clearly, we have come a long way in our understanding of the *Francisella* T6SS, yet much remains to be determined. Key unresolved issues include: (a) the protein composition and structure of the baseplate, (b) the intracellular signals that trigger contraction, (c) the receptors that sense these signals, (d) the signal transduction mechanism, (e) an atomic model of the baseplate and membrane complex, (f) the atomic structure of the extended sheath, and (g) identification of the functions of the secreted components and an understanding of how they promote phagosome escape and intracellular replication.

## Author contributions

All authors listed have made a substantial, direct and intellectual contribution to the work, and approved it for publication.

### Conflict of interest statement

The authors declare that the research was conducted in the absence of any commercial or financial relationships that could be construed as a potential conflict of interest.

## References

[B1] Aschtgen M. S. Gavioli M. Dessen A. Lloubès R. Cascales E. (2010). The SciZ protein anchors the enteroaggregative *Escherichia coli* Type VI secretion system to the cell wall. Mol. Microbiol. 75, 886–899. 10.1111/j.1365-2958.2009.07028.x 20487285

[B2] Bartual S. G. Otero J. M. Garcia-Doval C. Llamas-Saiz A. L. Kahn R. Fox G. C.. (2010). Structure of the bacteriophage T4 long tail fiber receptor-binding tip. Proc. Natl. Acad. Sci. U.S.A. 107, 20287–20292. 10.1073/pnas.1011218107 21041684 PMC2996694

[B3] Basler M. (2015). Type VI secretion system: secretion by a contractile nanomachine. Philos. Trans. R. Soc. Lond. B Biol. Sci. 370:20150021. 10.1098/rstb.2015.0021 26370934 PMC4632598

[B4] Basler M. Ho B. T. Mekalanos J. J. (2013). Tit-for-tat: type VI secretion system counterattack during bacterial cell-cell interactions. Cell 152, 884–894. 10.1016/j.cell.2013.01.042 23415234 PMC3616380

[B5] Basler M. Mekalanos J. J. (2012). Type 6 secretion dynamics within and between bacterial cells. Science 337, 815–815. 10.1126/science.1222901 22767897 PMC3557511

[B6] Basler M. Pilhofer M. Henderson G. P. Jensen G. J. Mekalanos J. J. (2012). Type VI secretion requires a dynamic contractile phage tail-like structure. Nature 483, 182–186. 10.1038/nature10846 22367545 PMC3527127

[B7] Bebeacua C. Lai L. Vegge C. S. Brøndsted L. Van Heel M. Veesler D.. (2013). Visualizing a complete Siphoviridae member by single-particle electron microscopy: the structure of lactococcal phage TP901-1. J. Virol. 87, 1061–1068. 10.1128/JVI.02836-12 23135714 PMC3554098

[B8] Bell J. F. Owen C. R. Larson C. L. (1955). Virulence of Bacterium tularense. I. A study of the virulence of Bacterium tularense in mice, guinea pigs, and rabbits. J. Infect. Dis. 97, 162–166. 10.1093/infdis/97.2.162 13263645

[B9] Bingle L. E. Bailey C. M. Pallen M. J. (2008). Type VI secretion: a beginner's guide. Curr. Opin. Microbiol. 11, 3–8. 10.1016/j.mib.2008.01.006 18289922

[B10] Bladergroen M. R. Badelt K. Spaink H. P. (2003). Infection-blocking genes of a symbiotic *Rhizobium leguminosarum* strain that are involved in temperature-dependent protein secretion. Mol. Plant Microbe Interact. 16, 53–64. 10.1094/MPMI.2003.16.1.53 12580282

[B11] Böck D. Medeiros J. M. Tsao H. F. Penz T. Weiss G. L. Aistleitner K.. (2017). *In situ* architecture, function, and evolution of a contractile injection system. Science 357, 713–717. 10.1126/science.aan7904 28818949 PMC6485382

[B12] Bourdonnay E. Henry T. (2016). Catch me if you can. Elife 5:e14721. 10.7554/eLife.14721 26919282 PMC4786409

[B13] Boyer F. Fichant G. Berthod J. Vandenbrouck Y. Attree I. (2009). Dissecting the bacterial type VI secretion system by a genome wide in silico analysis: what can be learned from available microbial genomic resources? BMC Genomics 10:104. 10.1186/1471-2164-10-104 19284603 PMC2660368

[B14] Brackmann M. Wang J. Basler M. (2018). Type VI secretion system sheath inter-subunit interactions modulate its contraction. EMBO Rep. 19, 225–233. 10.15252/embr.201744416 29222345 PMC5797969

[B15] Brodmann M. Dreier R. F. Broz P. Basler M. (2017). *Francisella* requires dynamic type VI secretion system and ClpB to deliver effectors for phagosomal escape. Nat. Commun. 8:15853. 10.1038/ncomms15853 28621333 PMC5481754

[B16] Bröms J. E. Lavander M. Meyer L. Sjostedt A. (2011). IglG and IglI of the *Francisella* pathogenicity island are important virulence determinants of *Francisella tularensis* LVS. Infect. Immun. 79, 3683–3696. 10.1128/IAI.01344-10 21690239 PMC3165494

[B17] Bröms J. E. Lavander M. Sjöstedt A. (2009). A conserved alpha-helix essential for a type VI secretion-like system of *Francisella tularensis*. J. Bacteriol. 191, 2431–2446. 10.1128/JB.01759-08 19201795 PMC2668417

[B18] Bröms J. E. Meyer L. Sun K. Lavander M. Sjostedt A. (2012). Unique substrates secreted by the type VI secretion system of *Francisella tularensis* during intramacrophage infection. PLoS ONE 7:e50473. 10.1371/journal.pone.0050473 23185631 PMC3502320

[B19] Bröms J. E. Sjöstedt A. Lavander M. (2010). The role of the *Francisella tularensis* pathogenicity island in Type VI secretion, intracellular survival, and modulation of host cell signaling. Front. Microbiol. 1:136. 10.3389/fmicb.2010.00136 21687753 PMC3109350

[B20] Brunet Y. R. Espinosa L. Harchouni S. Mignot T. Cascales E. (2013). Imaging type VI secretion-mediated bacterial killing. Cell Rep. 3, 36–41. 10.1016/j.celrep.2012.11.027 23291094

[B21] Brunet Y. R. Hénin J. Celia H. Cascales E. (2014). Type VI secretion and bacteriophage tail tubes share a common assembly pathway. EMBO Rep. 15, 315–321. 10.1002/embr.201337936 24488256 PMC3989698

[B22] Brunet Y. R. Zoued A. Boyer F. Douzi B. Cascales E. (2015). The Type VI secretion TssEFGK-VgrG phage-like baseplate is recruited to the TssJLM membrane complex via multiple contacts and serves as assembly platform for tail tube/sheath polymerization. PLoS Genet. 11:e1005545. 10.1371/journal.pgen.1005545 26460929 PMC4604203

[B23] Burtnick M. N. Brett P. J. Harding S. V. Ngugi S. A. Ribot W. J. Chantratita N.. (2011). The cluster 1 type VI secretion system is a major virulence determinant in *Burkholderia pseudomallei*. Infect. Immun. 79, 1512–1525. 10.1128/IAI.01218-10 21300775 PMC3067527

[B24] Chang Y. W. Rettberg L. A. Ortega D. R. Jensen G. J. (2017). *In vivo* structures of an intact type VI secretion system revealed by electron cryotomography. EMBO Rep. 18, 1090–1099. 10.15252/embr.201744072 28487352 PMC5494534

[B25] Checroun C. Wehrly T. D. Fischer E. R. Hayes S. F. Celli J. (2006). Autophagy-mediated reentry of *Francisella tularensis* into the endocytic compartment after cytoplasmic replication. Proc. Natl. Acad. Sci. U.S.A. 103, 14578–14583. 10.1073/pnas.0601838103 16983090 PMC1600002

[B26] Chiu H. C. Soni S. Kulp S. K. Curry H. Wang D. Gunn J. S.. (2009). Eradication of intracellular *Francisella tularensis* in THP-1 human macrophages with a novel autophagy inducing agent. J. Biomed. Sci. 16:110. 10.1186/1423-0127-16-110 20003180 PMC2801672

[B27] Chong A. Celli J. (2010). The *Francisella* intracellular life cycle: toward molecular mechanisms of intracellular survival and proliferation. Front. Microbiol. 1:138. 10.3389/fmicb.2010.00138 21687806 PMC3109316

[B28] Cianfanelli F. R. Alcoforado Diniz J. Guo M. De Cesare V. Trost M. Coulthurst S. J. (2016a). VgrG and PAAR proteins define distinct versions of a functional type VI secretion system. PLoS Pathog. 12:e1005735. 10.1371/journal.ppat.1005735 27352036 PMC4924876

[B29] Cianfanelli F. R. Monlezun L. Coulthurst S. J. (2016b). Aim, load, fire: the type VI secretion system, a bacterial nanoweapon. Trends Microbiol. 24, 51–62. 10.1016/j.tim.2015.10.005 26549582

[B30] Clemens D. L. Ge P. Lee B. Y. Horwitz M. A. Zhou Z. H. (2015). Atomic structure of T6SS reveals interlaced array essential to function. Cell 160, 940–951. 10.1016/j.cell.2015.02.005 25723168 PMC4351867

[B31] Clemens D. L. Horwitz M. A. (2007). Uptake and intracellular fate of *Francisella tularensis* in human macrophages. Ann. N. Y. Acad. Sci. 1105, 160–186. 10.1196/annals.1409.001 17435118

[B32] Clemens D. L. Lee B. Y. Horwitz M. A. (2004). Virulent and avirulent strains of *Francisella tularensis* prevent acidification and maturation of their phagosomes and escape into the cytoplasm in human macrophages. Infect. Immun. 72, 3204–3217. 10.1128/IAI.72.6.3204-3217.2004 15155622 PMC415696

[B33] Clemens D. L. Lee B. Y. Horwitz M. A. (2005). *Francisella tularensis* enters macrophages via a novel process involving pseudopod loops. Infect. Immun. 73, 5892–5902. 10.1128/IAI.73.9.5892-5902.2005 16113308 PMC1231130

[B34] Clemens D. L. Lee B. Y. Horwitz M. A. (2012). O-antigen-deficient *Francisella tularensis* Live Vaccine Strain mutants are ingested via an aberrant form of looping phagocytosis and show altered kinetics of intracellular trafficking in human macrophages. Infect. Immun. 80, 952–967. 10.1128/IAI.05221-11 22202123 PMC3294633

[B35] Das S. Chaudhuri K. (2003). Identification of a unique IAHP (IcmF associated homologous proteins) cluster in *Vibrio cholerae* and other proteobacteria through in silico analysis. In Silico Biol. 3, 287–300. 12954091

[B36] de Bruin O. M. Duplantis B. N. Ludu J. S. Hare R. F. Nix E. B. Schmerk C. L.. (2011). The biochemical properties of the *Francisella* pathogenicity island (FPI)-encoded proteins IglA, IglB, IglC, PdpB and DotU suggest roles in type VI secretion. Microbiology 157, 3483–3491. 10.1099/mic.0.052308-0 21980115 PMC3352279

[B37] de Bruin O. M. Ludu J. S. Nano F. E. (2007). The *Francisella* pathogenicity island protein IglA localizes to the bacterial cytoplasm and is needed for intracellular growth. BMC Microbiol. 7:1. 10.1186/1471-2180-7-1 17233889 PMC1794414

[B38] Decoin V. Barbey C. Bergeau D. Latour X. Feuilloley M. G. J. Orange N.. (2014). A type VI secretion system is involved in *Pseudomonas fluorescens* bacterial competition. PLoS ONE 9:e89411. 10.1371/journal.pone.0089411 24551247 PMC3925238

[B39] Deng K. Blick R. J. Liu W. Hansen E. J. (2006). Identification of *Francisella tularensis* genes affected by iron limitation. Infect. Immun. 74, 4224–4236. 10.1128/IAI.01975-05 16790797 PMC1489736

[B40] Durand E. Cambillau C. Cascales E. Journet L. (2014). VgrG, Tae, Tle, and beyond: the versatile arsenal of Type VI secretion effectors. Trends Microbiol. 22, 498–507. 10.1016/j.tim.2014.06.004 25042941

[B41] Durand E. Nguyen V. S. Zoued A. Logger L. Péhau-Arnaudet G. Aschtgen M.-S.. (2015). Biogenesis and structure of a type VI secretion membrane core complex. Nature 523, 555–560. 10.1038/nature14667 26200339

[B42] Durand E. Zoued A. Spinelli S. Watson P. J. Aschtgen M. S. Journet L.. (2012). Structural characterization and oligomerization of the TssL protein, a component shared by bacterial type VI and type IVb secretion systems. J. Biol. Chem. 287, 14157–14168. 10.1074/jbc.M111.338731 22371492 PMC3340138

[B43] Ellis J. Oyston P. C. Green M. Titball R. W. (2002). Tularemia. Clin. Microbiol. Rev. 15, 631–646. 10.1128/CMR.15.4.631-646.2002 12364373 PMC126859

[B44] Eshraghi A. Kim J. Walls A. C. Ledvina H. E. Miller C. N. Ramsey K. M.. (2016). Secreted effectors encoded within and outside of the *Francisella* pathogenicity island promote intramacrophage growth. Cell Host Microbe 20, 573–583. 10.1016/j.chom.2016.10.008 27832588 PMC5384264

[B45] Felisberto-Rodrigues C. Durand E. Aschtgen M. S. Blangy S. Ortiz-Lombardia M. Douzi B.. (2011). Towards a structural comprehension of bacterial type VI secretion systems: characterization of the TssJ-TssM complex of an *Escherichia coli* pathovar. PLoS Pathog. 7:e1002386. 10.1371/journal.ppat.1002386 22102820 PMC3213119

[B46] Ge P. Scholl D. Leiman P. G. Yu X. Miller J. F. Zhou Z. H. (2015). Atomic structures of a bactericidal contractile nanotube in its pre- and postcontraction states. Nat. Struct. Mol. Biol. 22, 377–382. 10.1038/nsmb.2995 25822993 PMC4445970

[B47] Golovliov I. Baranov V. Krocova Z. Kovarova H. Sjöstedt A. (2003). An attenuated strain of the facultative intracellular bacterium *Francisella tularensis* can escape the phagosome of monocytic cells. Infect. Immun. 71, 5940–5950. 10.1128/IAI.71.10.5940-5950.2003 14500514 PMC201066

[B48] Golovliov I. Ericsson M. Sandström G G. Tärnvik A. Sjöstedt A. (1997). Identification of proteins of *Francisella tularensis* induced during growth in macrophages and cloning of the gene encoding a prominently induced 23-kilodalton protein. Infect. Immun. 65, 2183–2189. 9169749 10.1128/iai.65.6.2183-2189.1997 PMC175301

[B49] Govan J. R. (1974). Studies on the pyocins of *Pseudomonas aeruginosa*: morphology and mode of action of contractile pyocins. J. Gen. Microbiol. 80, 1–15. 10.1099/00221287-80-1-1 4206804

[B50] Gray C. G. Cowley S. C. Cheung K. K. Nano F. E. (2002). The identification of five genetic loci of *Francisella novicida* associated with intracellular growth. FEMS Microbiol. Lett. 215, 53–56. 10.1111/j.1574-6968.2002.tb11369.x 12393200

[B51] Haapalainen M. Mosorin H. Dorati F. Wu R. F. Roine E. Taira S.. (2012). Hcp2, a secreted protein of the phytopathogen *Pseudomonas syringae* pv. tomato DC3000, is required for fitness for competition against bacteria and yeasts. J. Bacteriol. 194, 4810–4822. 10.1128/JB.00611-12 22753062 PMC3430304

[B52] Henry T. Brotcke A. Weiss D. S. Thompson L. J. Monack D. M. (2007). Type I interferon signaling is required for activation of the inflammasome during *Francisella* infection. J. Exp. Med. 204, 987–994. 10.1084/jem.20062665 17452523 PMC2118578

[B53] Heymann J. B. Bartho J. D. Rybakova D. Venugopal H. P. Winkler D. C. Sen A.. (2013). Three-dimensional structure of the toxin-delivery particle antifeeding prophage of *Serratia entomophila*. J. Biol. Chem. 288, 25276–25284. 10.1074/jbc.M113.456145 23857636 PMC3757192

[B54] Higerd T. B. Baechler C. A. Berk R. S. (1969). Morphological studies on relaxed and contracted forms of purified pyocin particles. J. Bacteriol. 98, 1378–1389. 4977989 10.1128/jb.98.3.1378-1389.1969 PMC315335

[B55] Ho B. T. Dong T. G. Mekalanos J. J. (2014). A view to a kill: the bacterial type VI secretion system. Cell Host Microbe 15, 9–21. 10.1016/j.chom.2013.11.008 24332978 PMC3936019

[B56] Hood R. D. Peterson S. B. Mougous J. D. (2017). From striking out to striking gold: discovering that type VI secretion targets bacteria. Cell Host Microbe 21, 286–289. 10.1016/j.chom.2017.02.001 28279332 PMC6404758

[B57] Johansson A. Celli J. Conlan W. Elkins K. L. Forsman M. Keim P. S.. (2010). Objections to the transfer of *Francisella novicida* to the subspecies rank of *Francisella tularensis*. Int. J. Syst. Evol. Microbiol. 60, 1718–1720. 10.1099/ijs.0.022830-0 20688748 PMC7442299

[B58] Jones D. T. Taylor W. R. Thornton J. M. (1992). The rapid generation of mutation data matrices from protein sequences. Comput. Appl. Biosci. 8, 275–282. 10.1093/bioinformatics/8.3.275 1633570

[B59] Journet L. Cascales E. (2016). The type VI secretion system in *Escherichia coli* and related species. EcoSal Plus 7, 1–20. 10.1128/ecosalplus.ESP-0009-2015 27223818 PMC11575709

[B60] Kudryashev M. Wang R. Y. Brackmann M. Scherer S. Maier T. Baker D.. (2015). Structure of the type VI secretion system contractile sheath. Cell 160, 952–962. 10.1016/j.cell.2015.01.037 25723169 PMC4359589

[B61] Kumar S. Stecher G. Tamura K. (2016). MEGA7: molecular evolutionary genetics analysis version 7.0 for bigger datasets. Mol. Biol. Evol. 33, 1870–1874. 10.1093/molbev/msw054 27004904 PMC8210823

[B62] Lai X. H. Golovliov I. Sjöstedt A. (2001). *Francisella tularensis* induces cytopathogenicity and apoptosis in murine macrophages via a mechanism that requires intracellular bacterial multiplication. Infect. Immun. 69, 4691–4694. 10.1128/IAI.69.7.4691-4694.2001 11402018 PMC98551

[B63] Lai X. H. Golovliov I. Sjöstedt A. (2004). Expression of IglC is necessary for intracellular growth and induction of apoptosis in murine macrophages by *Francisella tularensis*. Microb. Pathog. 37, 225–230. 10.1016/j.micpath.2004.07.002 15519043

[B64] Legrand P. Collins B. Blangy S. Murphy J. Spinelli S. Gutierrez C.. (2016). The atomic structure of the phage Tuc2009 baseplate tripod suggests that host recognition involves two different carbohydrate binding modules. mBio 7:e01781-15. 10.1128/mBio.01781-15 26814179 PMC4742702

[B65] Leiman P. G. Chipman P. R. Kostyuchenko V. A. Mesyanzhinov V. V. Rossmann M. G. (2004). Three-dimensional rearrangement of proteins in the tail of bacteriophage T4 on infection of its host. Cell 118, 419–429. 10.1016/j.cell.2004.07.022 15315755

[B66] Leiman P. G. Shneider M. M. (2012). Contractile tail machines of bacteriophages. Adv. Exp. Med. Biol. 726, 93–114. 10.1007/978-1-4614-0980-9_5 22297511

[B67] Lindgren H. Golovliov I. Baranov V. Ernst R. K. Telepnev M. Sjöstedt A. (2004). Factors affecting the escape of *Francisella tularensis* from the phagolysosome. J. Med. Microbiol. 53, 953–958. 10.1099/jmm.0.45685-0 15358816

[B68] Logger L. Aschtgen M. S. Guérin M. Cascales E. Durand E. (2016). Molecular dissection of the interface between the type VI secretion TssM cytoplasmic domain and the TssG baseplate component. J. Mol. Biol. 428, 4424–4437. 10.1016/j.jmb.2016.08.032 27600411

[B69] Long M. E. Lindemann S. R. Rasmussen J. A. Jones B. D. Allen L. A. (2013). Disruption of *Francisella tularensis* Schu S4 iglI, iglJ, and pdpC genes results in attenuation for growth in human macrophages and *in vivo* virulence in mice and reveals a unique phenotype for pdpC. Infect. Immun. 81, 850–861. 10.1128/IAI.00822-12 23275090 PMC3584877

[B70] Ludu J. S. de Bruin O. M. Duplantis B. N. Schmerk C. L. Chou A. Y. Elkins K. L.. (2008). The *Francisella* pathogenicity island protein PdpD is required for full virulence and associates with homologues of the type VI secretion system. J. Bacteriol. 190, 4584–4595. 10.1128/JB.00198-08 18469101 PMC2446798

[B71] Ma L. S. Lin J. S. Lai E. M. (2009). An IcmF family protein, ImpLM, is an integral inner membrane protein interacting with ImpKL, and its walker a motif is required for type VI secretion system-mediated Hcp secretion in *Agrobacterium tumefaciens*. J. Bacteriol. 191, 4316–4329. 10.1128/JB.00029-09 19395482 PMC2698499

[B72] Ma L. S. Narberhaus F. Lai E. M. (2012). IcmF family protein TssM exhibits ATPase activity and energizes type VI secretion. J. Biol. Chem. 287, 15610–15621. 10.1074/jbc.M111.301630 22393043 PMC3346141

[B73] Man S. M. Karki R. Malireddi R. K. Neale G. Vogel P. Yamamoto M.. (2015). The transcription factor IRF1 and guanylate-binding proteins target activation of the AIM2 inflammasome by *Francisella* infection. Nat. Immunol. 16, 467–475. 10.1038/ni.3118 25774715 PMC4406811

[B74] Mariathasan S. Weiss D. S. Dixit V. M. Monack D. M. (2005). Innate immunity against *Francisella tularensis* is dependent on the ASC/caspase-1 axis. J. Exp. Med. 202, 1043–1049. 10.1084/jem.20050977 16230474 PMC2213215

[B75] Meunier E. Wallet P. Dreier R. F. Costanzo S. Anton L. Rühl S.. (2015). Guanylate-binding proteins promote activation of the AIM2 inflammasome during infection with *Francisella novicida*. Nat. Immunol. 16, 476–484. 10.1038/ni.3119 25774716 PMC4568307

[B76] Mougous J. D. Cuff M. E. Raunser S. Shen A. Zhou M. Gifford C. A.. (2006). A virulence locus of *Pseudomonas aeruginosa* encodes a protein secretion apparatus. Science 312, 1526–1530. 10.1126/science.1128393 16763151 PMC2800167

[B77] Murch A. L. Skipp P. J. Roach P. L. Oyston P. C. F. (2017). Whole genome transcriptomics reveals global effects including up-regulation of *Francisella* pathogenicity island gene expression during active stringent response in the highly virulent *Francisella tularensis* subsp. tularensis SCHU S4. Microbiology 163, 1664–1679. 10.1099/mic.0.000550 29034854 PMC5845702

[B78] Nano F. E. Zhang N. Cowley S. C. Klose K. E. Cheung K. K. Roberts M. J.. (2004). A *Francisella tularensis* pathogenicity island required for intramacrophage growth. J. Bacteriol. 186, 6430–6436. 10.1128/JB.186.19.6430-6436.2004 15375123 PMC516616

[B79] Nazarov S. Schneider J. P. Brackmann M. Goldie K. N. Stahlberg H. Basler M. (2018). Cryo-EM reconstruction of type VI secretion system baseplate and sheath distal end. EMBO J. 37:e97103. 10.15252/embj.201797103 29255010 PMC5813253

[B80] Nguyen J. Q. Gilley R. P. Zogaj X. Rodriguez S. A. Klose K. E. (2014). Lipidation of the FPI protein IglE contributes to *Francisella tularensis* ssp. novicida intramacrophage replication and virulence. Pathog. Dis. 72, 10–18. 10.1111/2049-632X.12167 24616435 PMC4160424

[B81] Nguyen V. S. Logger L. Spinelli S. Legrand P. Huyen Pham T. T. Nhung Trinh T. T.. (2017). Type VI secretion TssK baseplate protein exhibits structural similarity with phage receptor-binding proteins and evolved to bind the membrane complex. Nat. Microbiol. 2:17103. 10.1038/nmicrobiol.2017.103 28650463

[B82] Pell L. G. Kanelis V. Donaldson L. W. Lynne Howell P. L. Davidson A. R. (2009). The phage λ major tail protein structure reveals a common evolution for long-tailed phages and the type VI bacterial secretion system. Proc. Natl. Acad. Sci. U.S.A. 106, 4160–4165. 10.1073/pnas.0900044106 19251647 PMC2657425

[B83] Pettersen E. F. Goddard T. D. Huang C. C. Couch G. S. Greenblatt D. M. Meng E. C.. (2004). UCSF Chimera—a visualization system for exploratory research and analysis. J. Comput. Chem. 25, 1605–1612. 10.1002/jcc.20084 15264254

[B84] Pietrosiuk A. Lenherr E. D. Falk S. Bönemann G. Kopp J. Zentgraf H.. (2011). Molecular basis for the unique role of the AAA+ chaperone ClpV in type VI protein secretion. J. Biol. Chem. 286, 30010–30021. 10.1074/jbc.M111.253377 21733841 PMC3191042

[B85] Planamente S. Salih O. Manoli E. Albesa-Jové D. Freemont P. S. Filloux A. (2016). TssA forms a gp6-like ring attached to the type VI secretion sheath. EMBO J. 35, 1613–1627. 10.15252/embj.201694024 27288401 PMC4969574

[B86] Pukatzki S. Ma A. T. Revel A. T. Sturtevant D. Mekalanos J. J. (2007). Type VI secretion system translocates a phage tail spike-like protein into target cells where it cross-links actin. Proc. Natl. Acad. Sci. U.S.A. 104, 15508–15513. 10.1073/pnas.0706532104 17873062 PMC2000545

[B87] Pukatzki S. Ma A. T. Sturtevant D. Krastins B. Sarracino D. Nelson W. C.. (2006). Identification of a conserved bacterial protein secretion system in *Vibrio cholerae* using the Dictyostelium host model system. Proc. Natl. Acad. Sci. U.S.A. 103, 1528–1533. 10.1073/pnas.0510322103 16432199 PMC1345711

[B88] Rao P. S. Yamada Y. Tan Y. P. Leung K. Y. (2004). Use of proteomics to identify novel virulence determinants that are required for *Edwardsiella tarda* pathogenesis. Mol. Microbiol. 53, 573–586. 10.1111/j.1365-2958.2004.04123.x 15228535

[B89] Rao V. A. Shepherd S. M. English G. Coulthurst S. J. Hunter W. N. (2011). The structure of *Serratia marcescens* Lip, a membrane-bound component of the type VI secretion system. Acta Crystallogr. D Biol. Crystallogr. 67, 1065–1072. 10.1107/S0907444911046300 22120744 PMC3225178

[B90] Rigard M. Bröms J. E. Mosnier A. Hologne M. Martin A. Lindgren L.. (2016). *Francisella tularensis* IglG belongs to a novel family of PAAR-like T6SS proteins and harbors a unique N-terminal extension required for virulence. PLoS Pathog. 12:e1005821. 10.1371/journal.ppat.1005821 27602570 PMC5014421

[B91] Robb C. S. Nano F. E. Boraston A. B. (2012). The structure of the conserved type six secretion protein TssL (DotU) from *Francisella novicida*. J. Mol. Biol. 419, 277–283. 10.1016/j.jmb.2012.04.003 22504227

[B92] Robertson G. T. Child R. Ingle C. Celli J. Norgard M. V. (2013). IglE is an outer membrane-associated lipoprotein essential for intracellular survival and murine virulence of type A *Francisella tularensis*. Infect. Immun. 81, 4026–4040. 10.1128/IAI.00595-13 23959721 PMC3811846

[B93] Russell A. B. Peterson S. B. Mougous J. D. (2014a). Type VI secretion system effectors: poisons with a purpose. Nat. Rev. Microbiol. 12, 137–148. 10.1038/nrmicro3185 24384601 PMC4256078

[B94] Russell A. B. Wexler A. G. Harding B. N. Whitney J. C. Bohn A. J. Goo Y. A.. (2014b). A type VI secretion-related pathway in *Bacteroidetes* mediates interbacterial antagonism. Cell Host Microbe 16, 227–236. 10.1016/j.chom.2014.07.007 25070807 PMC4136423

[B95] Sana T. G. Berni B. Bleves S. (2016). The T6SSs of *Pseudomonas aeruginosa* Strain PAO1 and Their effectors: beyond bacterial-cell targeting. Front. Cell. Infect. Microbiol. 6:61. 10.3389/fcimb.2016.00061 27376031 PMC4899435

[B96] Sana T. G. Hachani A. Bucior I. Soscia C. Garvis S. Termine E.. (2012). The second type VI secretion system of *Pseudomonas aeruginosa* strain PAO1 is regulated by quorum sensing and Fur and modulates internalization in epithelial cells. J. Biol. Chem. 287, 27095–27105. 10.1074/jbc.M112.376368 22665491 PMC3411052

[B97] Santin Y. G. Cascales E. (2017). Domestication of a housekeeping transglycosylase for assembly of a Type VI secretion system. EMBO Rep. 18, 138–149. 10.15252/embr.201643206 27920034 PMC5210162

[B98] Saslaw S. Eigelsbach H. T. Prior J. A. Wilson H. E. Carhart S. (1961a). Tularemia vaccine study. I: intracutaneous challenge. Arch. Intern. Med. 107, 121–133. 10.1001/archinte.1961.03620050055006 13746668

[B99] Saslaw S. Eigelsbach H. T. Prior J. A. Wilson H. E. Carhart S. (1961b). Tularemia vaccine study. II. respiratory challenge. Arch. Intern. Med. 107, 702–714. 10.1001/archinte.1961.03620050068007 13746667

[B100] Schwarz S. West T. E. Boyer F. Chiang W. C. Carl M. A. Hood R. D.. (2010). Burkholderia type VI secretion systems have distinct roles in eukaryotic and bacterial cell interactions. PLoS Pathog. 6:e1001068. 10.1371/journal.ppat.1001068 20865170 PMC2928800

[B101] Shikuma N. J. Pilhofer M. Weiss G. L. Hadfield M. G. Jensen G. J. Newman D. K. (2014). Marine tubeworm metamorphosis induced by arrays of bacterial phage tail–like structures. Science 343, 529–533. 10.1126/science.1246794 24407482 PMC4949041

[B102] Shneider M. M. Buth S. A. Ho B. T. Basler M. Mekalanos J. J. Leiman P. G. (2013). PAAR-repeat proteins sharpen and diversify the type VI secretion system spike. Nature 500, 350–353. 10.1038/nature12453 23925114 PMC3792578

[B103] Silverman J. M. Agnello D. M. Zheng H. Andrews B. T. Li M. Catalano C. E.. (2013). Haemolysin coregulated protein is an exported receptor and chaperone of type VI secretion substrates. Mol. Cell 51, 584–593. 10.1016/j.molcel.2013.07.025 23954347 PMC3844553

[B104] Steele S. Radlinski L. Taft-Benz S. Brunton J. Kawula T. H. (2016). Trogocytosis-associated cell to cell spread of intracellular bacterial pathogens. Elife 5:e10625. 10.7554/eLife.10625 26802627 PMC4786427

[B105] Suarez G. Sierra J. C. Erova T. E. Sha J. Horneman A. J. Chopra A. K. (2010). A type VI secretion system effector protein, VgrG1, from *Aeromonas hydrophila* that induces host cell toxicity by ADP ribosylation of actin. J. Bacteriol. 192, 155–168. 10.1128/JB.01260-09 19880608 PMC2798274

[B106] Suarez G. Sierra J. C. Sha J. Wang S. Erova T. E. Fadl A. A.. (2008). Molecular characterization of a functional type VI secretion system from a clinical isolate of *Aeromonas hydrophila*. Microb. Pathog. 44, 344–361. 10.1016/j.micpath.2007.10.005 18037263 PMC2430056

[B107] Sun P. Austin B. P. Schubot F. D. Waugh D. S. (2007). New protein fold revealed by a 1.65 A resolution crystal structure of *Francisella tularensis* pathogenicity island protein IglC. Protein Sci. 16, 2560–2563. 10.1110/ps.073177307 17905833 PMC2211698

[B108] Taylor N. M. Prokhorov N. S. Guerrero-Ferreira R. C. Shneider M. M. Browning C. Goldie K. N.. (2016). Structure of the T4 baseplate and its function in triggering sheath contraction. Nature 533, 346–352. 10.1038/nature17971 27193680

[B109] Uda A. Sekizuka T. Tanabayashi K. Fujita O. Kuroda M. Hotta A.. (2014). Role of pathogenicity determinant protein C (PdpC) in determining the virulence of the *Francisella tularensis* subspecies tularensis SCHU. PLoS ONE 9:e89075. 10.1371/journal.pone.0089075 24558472 PMC3928404

[B110] Wang J. Brackmann M. Castaño-Díez D. Kudryashev M. Goldie K. N. Maier T.. (2017). Cryo-EM structure of the extended type VI secretion system sheath–tube complex. Nat. Microbiol. 2, 1507–1512. 10.1038/s41564-017-0020-7 28947741

[B111] Weber B. S. Hennon S. W. Wright M. S. Scott N. E. de Berardinis V. Foster L. J.. (2016). Genetic dissection of the type VI secretion system in *Acinetobacter* and identification of a novel peptidoglycan hydrolase, TagX, required for its biogenesis. MBio 7:e01253-16. 10.1128/mBio.01253-16 27729508 PMC5061870

[B112] Wehrly T. D. Chong A. Virtaneva K. Sturdevant D. E. Child R. Edwards J. A.. (2009). Intracellular biology and virulence determinants of *Francisella tularensis* revealed by transcriptional profiling inside macrophages. Cell. Microbiol. 11, 1128–1150. 10.1111/j.1462-5822.2009.01316.x 19388904 PMC2746821

[B113] Weiss D. S. Brotcke A. Henry T. Margolis J. J. Chan K. Monack D. M. (2007a). *In vivo* negative selection screen identifies genes required for Francisella virulence. Proc. Natl. Acad. Sci. U.S.A. 104, 6037–6042. 10.1073/pnas.0609675104 17389372 PMC1832217

[B114] Weiss D. S. Henry T. Monack D. M. (2007b). *Francisella tularensis*: activation of the inflammasome. Ann. N. Y. Acad. Sci. 1105, 219–237. 10.1196/annals.1409.005 17395724

[B115] Whitney J. C. Beck C. M. Goo Y. A. Russell A. B. Harding B. N. De Leon J. A.. (2014). Genetically distinct pathways guide effector export through the type VI secretion system. Mol. Microbiol. 92, 529–542. 10.1111/mmi.12571 24589350 PMC4049467

[B116] Williams S. G. Varcoe L. T. Attridge S. R. Manning P. A. (1996). *Vibrio cholerae* Hcp, a secreted protein coregulated with HlyA. Infect. Immun. 64, 283–289. 8557353 10.1128/iai.64.1.283-289.1996 PMC173757

[B117] Zheng and Leung K. Y. (2007). Dissection of a type VI secretion system in *Edwardsiella tarda*. Mol. Microbiol. 66, 1192–1206. 10.1111/j.1365-2958.2007.05993.x 17986187

[B118] Zoued A. Cassaro C. J. Durand E. Douzi B. España A. P. Cambillau C.. (2016a). Structure-function analysis of the TssL cytoplasmic domain reveals a new interaction between the type VI secretion baseplate and membrane complexes. J. Mol. Biol. 428, 4413–4423. 10.1016/j.jmb.2016.08.030 27600409

[B119] Zoued A. Durand E. Bebeacua C. Brunet Y. R. Douzi B. Cambillau C.. (2013). TssK is a trimeric cytoplasmic protein interacting with components of both phage-like and membrane anchoring complexes of the type VI secretion system. J. Biol. Chem. 288, 27031–27041. 10.1074/jbc.M113.499772 23921384 PMC3779704

[B120] Zoued A. Durand E. Brunet Y. R. Spinelli S. Douzi B. Guzzo M.. (2016b). Priming and polymerization of a bacterial contractile tail structure. Nature 531, 59–63. 10.1038/nature17182 26909579

[B121] Zoued A. Durand E. Santin Y. G. Journet L. Roussel A. Cambillau C.. (2017). TssA: the cap protein of the type VI secretion system tail. Bioessays 39:1600262. 10.1002/bies.201600262 28817192

